# Molecular insights into photosynthesis and carbohydrate metabolism in *Jatropha curcas* grown under elevated CO_2_ using transcriptome sequencing and assembly

**DOI:** 10.1038/s41598-017-11312-y

**Published:** 2017-09-11

**Authors:** Sumit Kumar, Rachapudi Venkata Sreeharsha, Shalini Mudalkar, Prasad M. Sarashetti, Attipalli Ramachandra Reddy

**Affiliations:** 1 Photosynthesis and Stress Biology Laboratory, Department of Plant Sciences, University of Hyderabad, Hyderabad, India; 2 Genotypic Technology Private Limited, Bangalore, India

## Abstract

*Jatropha curcas* L. (Family – Euphorbiaceae) is a perennial tree of special interest due to its potential as a biofuel plant with high carbon sequestration. In this study, physiological investigations coupled with transcriptomics in relation to photosynthesis were evaluated in *Jatropha* grown under ambient (395 ppm) and elevated (550 ppm) CO_2_ atmosphere. Morphophysiological analysis revealed that *Jatropha* sustained enhanced photosynthesis during its growth under elevated CO_2_ for one year which might be linked to improved CO_2_ assimilation physiology and enhanced sink activity. We sequenced and analyzed the leaf transcriptome of *Jatropha* after one year of growth in both conditions using Illumina HiSeq platform. After optimized assembly, a total of 69,581 unigenes were generated. The differential gene expression (DGE) analysis revealed 3013 transcripts differentially regulated in elevated CO_2_ conditions. The photosynthesis regulatory genes were analysed for temporal expression patterns at four different growth phases which highlighted probable events contributing to enhanced growth and photosynthetic capacity including increased reducing power, starch synthesis and sucrose mobilization under elevated CO_2_. Overall, our data on physiological and transcriptomic analyses suggest an optimal resource allocation to the available and developing sink organs thereby sustaining improved photosynthetic rates during long-term growth of *Jatropha* under CO_2_ enriched environment.

## Introduction

Atmospheric carbon dioxide concentration [CO_2_] has increased from 280 μmol mol^−1^ to current 395 μmol mol^−1^ since the pre-industrial era and is expected to reach between 500–900 μmol mol^−1^ by the end of this century^1^. The adaptive capacity of plants to increased [CO_2_] in atmosphere depends on a range of physiological processes which varies among different plant species. Earlier studies have reported that the initial stimulation in photosynthetic potential under prolonged exposure to elevated [CO_2_] was followed by an acclimatory down-regulation in photosynthesis leading to less than predicted yields in many plant species^2, 3^. However, some fast growing coppice plantations with high yield potential have been able to sustain increased growth and productivity under elevated [CO_2_] over a long period of time suggesting that maintenance of short rotation plantations is an effective strategy for mitigation of increasing atmospheric [CO_2_] proportionally^4, 5^. The rapid growth of these trees also allows the analysis of their responses to elevated [CO_2_] over a complete production cycle^6^. The ability of such plants to sense and respond to the elevated CO_2_ environment requires transcriptional cascades operating at cellular level to adjust their morphology, physiology and phenotype accordingly^7^. Hence, studying the molecular mechanisms associated with growth of these trees to predicted elevated [CO_2_] can provide an insight into certain key candidate genes and the pathways controlled by them in order to understand the relationship between gene expression and adaptation to varying external environments.

The depletion of fossil fuel reserves and anthropogenic increase in emission of greenhouse gases have developed worldwide interest in renewable sources of energy including biofuels obtained from both carbohydrate- or oil-based feedstock and biomass. *J. curcas*, a member of family *Euphorbiaceae*, has been advocated as a plant with high potential for biofuel plantations because of its high seed oil content, easy propagation, rapid growth, short gestation period and adaptation to a wide range of agro-climatic conditions^8^. Apart from its usage in biofuels, bioenergy cropping also has an additional advantage of increasing the green cover of planet and sequestering excess carbon from atmosphere through photosynthesis thereby partially affecting the commitments of the Kyoto protocol^9^. It would be noteworthy to understand the molecular aspects associated with growth and productivity in *Jatropha* in different growth conditions which is crucial in domesticating and developing cultivars for a wide variety of applications^10^.

Previous investigations on molecular responses in plants under elevated CO_2_ have been limited to small plants with only one or two studies undertaken on tree species. The studies reported on the responses of tree species to elevated CO_2_ reveal insights about secondary metabolism during delayed senescence and enhanced radial growth^11–13^. Recent advancements in next generation sequencing technologies like Solexa/Illumina platform based *de novo* RNA-sequencing and high throughput deep sequencing have allowed discovery of new genes, analysis of specific transcripts, gene expression and generation of transcript sequences of non-model organisms^14^. This approach has accelerated better understanding of complex transcriptional patterns and measurements of gene expression in different tissues or at different stages of plant development in response to varying external environments. Realizing its importance as an economic plant, it is only recently a few genomic resources have been generated for *Jatropha* ^15, 16^. However, studies on transcriptome analysis of *Jatropha* were limited to regulatory aspects of oil biosynthesis in developing seeds with little emphasis on growth and photosynthesis^17, 18^. Furthermore, few transcriptome studies, reported for growth of *Jatropha* under different environmental conditions, were limited to seedlings or pot-grown plants with no reports on plants grown for longer durations in field conditions^19–21^.

We have earlier reported growth, yield responses and carbon sequestration in *J. curcas* grown for two complete production cycles under elevated CO_2_ (~550 ppm) which demonstrated that *Jatropha* was able to sustain enhanced levels of photosynthesis throughout an year of growth in elevated CO_2_ atmosphere resulting in increased fruit and seed yields^22^. In this study, we performed RNA-seq analysis to explore the transcriptional variations in leaves of *Jatropha curcas* grown under ambient and elevated CO_2_ conditions. The leaf transcriptome of *Jatropha*, which was grown for a year under ambient and elevated CO_2_ environment were sequenced using Illumina technology and elevated CO_2_-responsive differentially expressed genes were identified. We integrated these findings with the morphophysiological data which was recorded continuously for the year comprising two growth seasons. This study is first of its kind on the transcriptomic analysis of *J. curcas* grown and maintained in field under elevated CO_2_ environment and also provides temporal expression pattern of certain crucial genes involved in photosynthesis and carbohydrate metabolism.

## Results

### Morphophysiological and foliar biochemical analysis of *Jatropha* under elevated CO_2_

**Figure 1:**
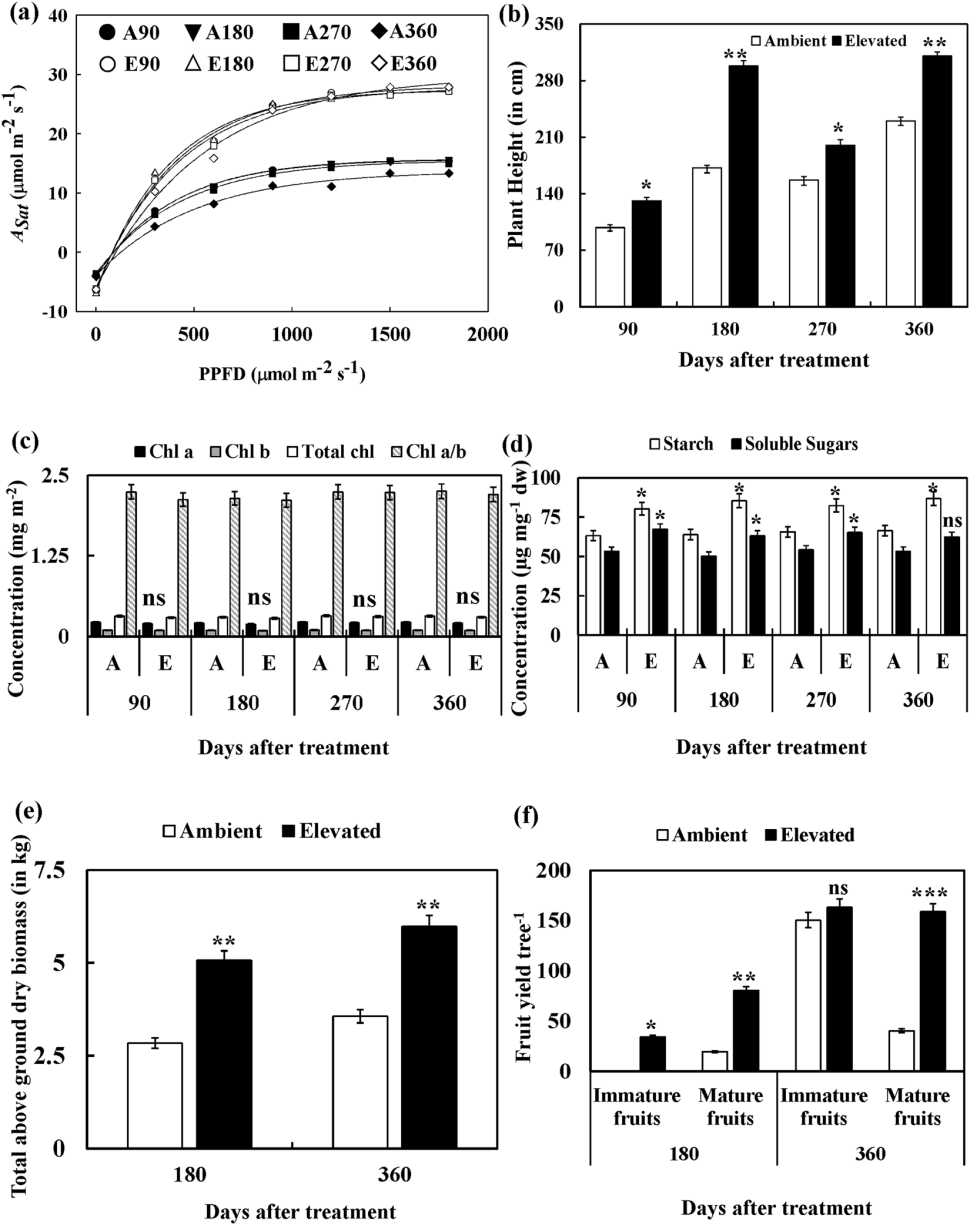
Comparative morphophysiology and biochemistry of *Jatropha* plants grown in ambient and elevated CO_2_ conditions after 90, 180, 270 and 360 days of growth comprising two growth seasons. (**a**) Photosynthetic A vs. Q Curve measured at saturating light intensities of 0, 300, 600, 900, 1200, 1500, 1800 and 2000 μmol photons m^−2^ s^−1^ at 90, 180, 270 and 360 days. (**b**) Plant height at 90, 180, 270 and 360 days of growth. (**c**) Chlorophyll content and Chl a/b in leaves of *Jatropha*. (**d**) Variations observed in the starch and soluble sugar content. (**e**) Above ground biomass after 180 and 360 days of growth. (**f**) Fruit yield per tree after 180 and 360 days of growth. The data given here represents average of six representative plants from two OTCs and values are expressed as mean ± SD. [Note – ns, not significant; *P < 0.05; **P < 0.01; ***P < 0.001].

Morphophysiological and biochemical variations were monitored during growth of *Jatropha* under ambient and elevated CO_2_ conditions at regular intervals for both seasons. There was no seasonal variation in most of the recorded parameters with a significant variation observed in reproductive yield for both growth seasons. The light-saturated photosynthetic rate (*A*_*sat*_) and apparent quantum efficiency (AQE; calculated as an initial slope of *A/Q* curve) were recorded to be significantly higher (~28 µmol m^−2^ s^−1^; ~0.030) at all four time points under elevated CO_2_ in comparison to ambient CO_2_ grown plants (~18 µmol m^−2^ s^−1^; ~0.020) which was sustained during both seasons (*P* < 0.01) (Fig. 1a, Table 1). Similarly, elevated CO_2_ grown *Jatropha* showed significant variations in chlorophyll a fluorescence characteristics in comparison to ambient grown plants (Table 1). The maximal photochemical efficiency of photosystem II (*F*_*v*_/*F*_*m*_), efficiency of water splitting complex (*F*_*v*_/*F*_*o*_), electron transport rate (*ETR*), effective quantum yield of PS II (*ΔF*/*F*_*m*_′) and photochemical quenching (qP) for elevated CO_2_ grown plants were recorded to be ~10, ~40, ~10, ~15 and ~12% respectively, higher than ambient grown *Jatropha* plants (*P* < 0.05) during its growth in both seasons (Table 1). However, non-photochemical quenching (NPQ) was significantly decreased by ~25% in elevated CO_2_ grown *Jatropha* in comparison to ambient grown plants (*P* < 0.05) (Table 1). Further, *Jatropha* plants were able to sustain enhanced growth in elevated CO_2_ environment as demonstrated by the plant height, number of secondary and tertiary branches, and more number of flowers (Fig. 1b, Supplementary Figs S1, S2 and S3). At the end of both seasons, after 180 and 360 days of growth, the elevated CO_2_ grown plants reached the height of ~3 m in comparison to ambient grown plants (*P* < 0.01). However, the chlorophyll content (both chlorophyll a and b) did not show any significant variation at 90 and 270 days with a non-significant decrease after 180 and 360 days recorded under elevated CO_2_ conditions in comparison to ambient CO_2_ grown *Jatropha* plants (Fig. 1c). The Chl a/b ratio also was unaltered (~2–3) suggesting optimum photosynthesis. Among the foliar carbohydrates, both starch and soluble sugars were recorded to be significantly higher (*P* < 0.05) at all the four time points in elevated CO_2_ grown *Jatropha* plants in comparison to ambient CO_2_ grown plants (Fig. 1d). The above ground dry biomass (comprising leaf, stem and fruits) at the end of both seasons, 180 and 360 days after growth, was found to be significantly higher (*P* < 0.01; ~75% and ~67% respectively for both seasons) for elevated CO_2_ grown *Jatropha* plants (Fig. 1e). The most interesting observation was recorded in fruit yield of *Jatropha* with very little yield (~20) in ambient CO_2_ conditions at the end of first growth season (180 days) in comparison to elevated CO_2_ grown plants which recorded almost ~3 fold more yields (*P* < 0.01). However, the yield significantly improved in second growth season (360 days) for ambient grown plants (~190) but again the elevated CO_2_ grown *Jatropha* plants demonstrated better yield performance with a significant (*P* < 0.01) increase of ~1.5 folds (Fig. 1f). As the *Jatropha* plants showed better photosynthetic performance in relation to source sink interaction under elevated CO_2_ for both seasons, we decided to sequence its transcriptome to identify probable molecular events in relation to photosynthetic physiology.

**Table 1:** Chlorophyll *a* fluorescence measurements on leaves of *Jatropha curcas* exposed to ambient (A) and elevated (E) [CO_2_] at 90, 180, 270 and 360 days.

Parameter	90 d		180 d		270 d		360 d	
	A	E	A	E	A	E	A	E
AQE	0.022 ± 0.002	0.032 ± 0.001^*^	0.021 ± 0.001	0.030 ± 0.001^*^	0.21 ± 0.001	0.030 ± 0.003^*^	0.020 ± 0.002	0.031 ± 0.003^*^
*F*_*v*_ */F*_*m*_	0.724 ± 0.05	0.804 ± 0.03^*^	0.718 ± 0.04	0.789 ± 0.05^*^	0.728 ± 0.05	0.804 ± 0.02^*^	0.724 ± 0.02	0.803 ± 0.04^*^
*F*_*v*_ */F*_*o*_	2.625 ± 0.46	3.64 ± 0.38^**^	2.634 ± 0.35	3.713 ± 0.35^**^	2.768 ± 0.45	3.930 ± 0.45^**^	2.492 ± 0.25	3.488 ± 0.36^**^
*ΔF*/*F*_*m*_′	0.685 ± 0.05	0.754 ± 0.05^*^	0.680 ± 0.03	0.748 ± 0.03^*^	0.692 ± 0.01	0.761 ± 0.02^*^	0.693 ± 0.02	0.762 ± 0.05^*^
ETR	148.4 ± 4.52	161.4 ± 7.24^*^	146.4 ± 3.05	168.3 ± 5.05^*^	152.5 ± 2.26	175.3 ± 4.25^*^	150.6 ± 4.29	173.1 ± 4.38^*^
NPQ	0.589 ± 0.05	0.464 ± 0.05^*^	0.575 ± 0.04	0.431 ± 0.02^*^	0.572 ± 0.05	0.429 ± 0.01^*^	0.574 ± 0.03	0.419 ± 0.02^*^
qP	0.726 ± 0.03	0.801 ± 0.02^*^	0.718 ± 0.02	0.825 ± 0.03^*^	0.728 ± 0.05	0.815 ± 0.03^*^	0.730 ± 0.04	0.817 ± 0.05^*^

Values were mean ± SD (*n* = 6), followed by significance of difference under elevated CO_2_ [*P < 0.05; **P < 0.01].

### Sequencing and transcript assembly

Sequencing of constructed cDNA library resulted in generation of 101 bp raw reads of fastq file size of 9.98 (42.69 millions) and 9.56 GB (40.84 millions) for ambient (sample A) and elevated (sample E) respectively (Supplementary Table S1). More than 85% of high quality (HQ) reads with average Phred quality score of ≥30 at each base position (Supplementary Fig. S4) were obtained and used for downstream analyses. A total of 44,179 and 28,825 contigs with maximum contig length of 8302 and 6817 were acquired for sample A and E, respectively. A total of 61,779 and 48,775 transcripts were obtained having a maximum transcript length 10,713 and 9,793 and a N50 value of 2304 and 2032 for sample A and E respectively (Supplementary Table S2). Also, the assembled transcriptome of *Jatropha* demonstrated ≥90% similarity with *Jatropha* genome (Supplementary Table S3). An average of 97.86% of reads matched to the reference genome covering ~87.7% of gene models/~89% of transcripts. An average of 92% of reads completely matched gene model indicating 5% of the transcript reads are matched to the non-gene model position of the genome which may be novel transcripts identified in our study. A total of 1809 (sample A) and 808 (sample E) transcripts generated from unaligned reads to genome (Supplementary Table S3). A total of 69,581 unigenes having 95% identity were obtained after primary analysis and clustering with CD-HIT with an average unigene length of 1657 and N50 value of 2225 (Table 2).

**Table 2:** Unigene statistics of *Jatropha curcas* L. transcriptome under elevated CO_2_.

Number of Transcripts Identified	69581
Maximum Contig Length	10713
Minimum Contig Length	200
Average Contig Length	1,657.4 ± 1,136.6
Median Contig Length	425
Total Contigs Length	11,53,25,317
Total Number of Non-ATGC Characters	3425
Percentage of Non-ATGC Characters	0.003
Contigs > = 200 bp	69581
Contigs > = 500 bp	58502
Contigs > = 1 Kbp	46868
Contigs > = 10 Kbp	2
N50 Value	2225

### Functional annotation and differential gene expression analysis

An expression matrix of gene-wise FPKM summary was prepared for reference assembled reads (Supplementary Table S4). Among the *de novo* assembled unigenes, a total of 9,562 unigenes were found to be differentially expressed in elevated CO_2_ in comparison to ambient grown *Jatropha* plants (Supplementary Table S5, Supplementary Fig. S5). Among them, 3672 unigenes were up-regulated and 5890 unigenes were down-regulated in elevated CO_2_ grown *Jatropha* plants. Furthermore, on the basis of applied yardstick for selecting differential expressed genes (DEGs) [*P* < 0.05, FDR < 0.01 and −1.00 ≥ |log_2_foldchange| ≥ 1.00], a total of 3013 unigenes were differentially expressed in response to CO_2_ treatment in leaves which comprised 833 (~27%) up-regulated unigenes and 2180 (~73%) down-regulated unigenes (Supplementary Table S6). The majority of Gene Ontology (GO) terms assigned to unigenes belonged to ATP binding in molecular function, integral to membrane in cellular functions and carbohydrate metabolic process in biological function category respectively (Supplementary Fig. S6a). A total of 27,125 (~38%) unigenes from ambient and elevated CO_2_-grown *Jatropha* were annotated based on EuKaryotic Orthologous Group (KOG) classification (Supplementary Fig. S6b). Further, 6568 unigenes were assigned to 275 known pathways. The majority of annotated transcripts were associated to carbohydrate metabolism (~14.98%) followed by translation (~12.46%), protein folding, sorting and degradation (~10.65%) and amino acid metabolism (~9.08%) (Supplementary Fig. S7).

**Figure 2:**
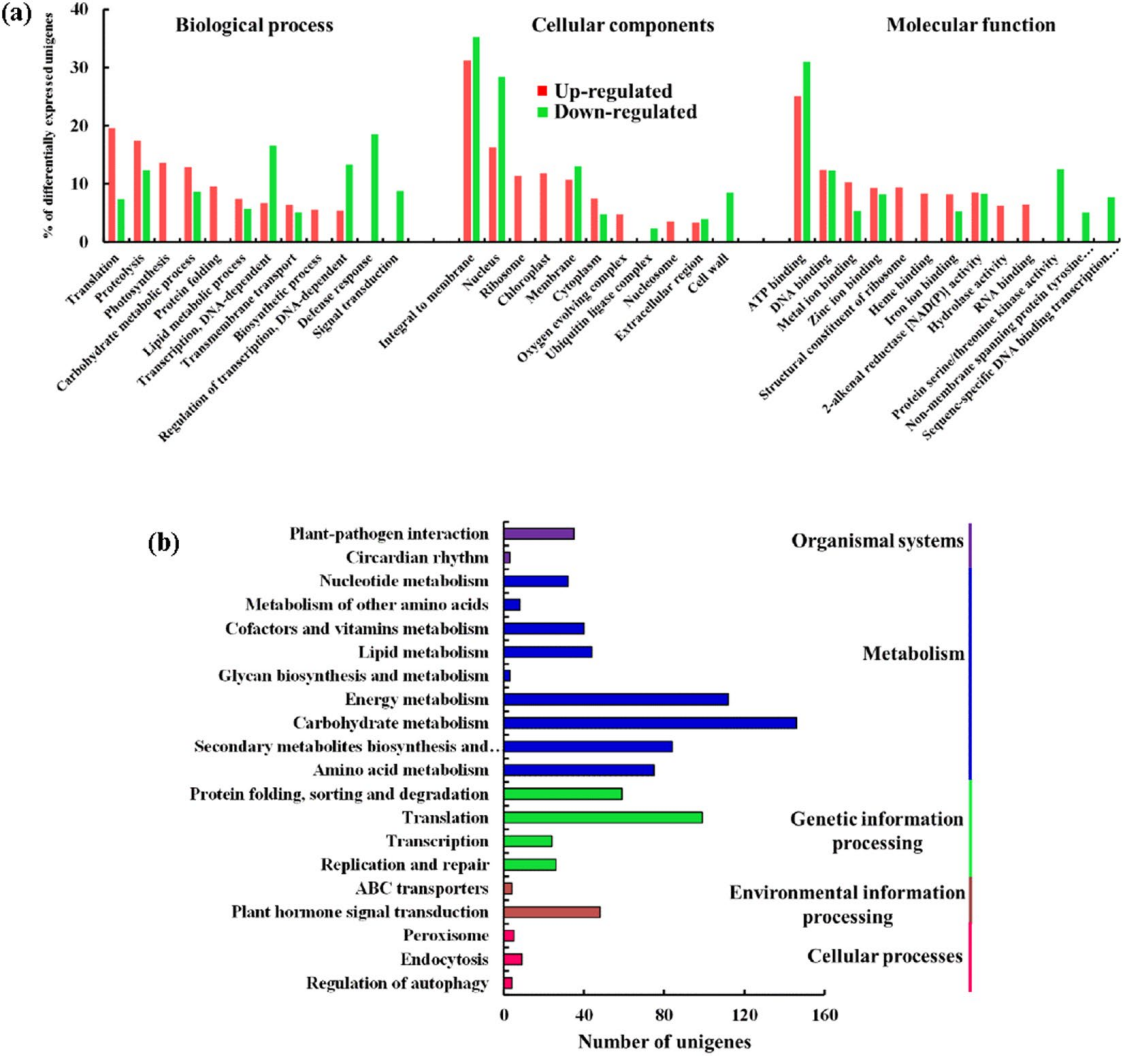
Enriched GO terms and KEGG classifications of the differentially expressed unigenes in leaves of elevated CO_2_-grown *J*. *curcas*. (**a**) The GO terms are categorized into ‘Molecular function’, ‘Cellular component’ and ‘Biological pathway’ with the percentage of unigenes up-regulated depicted in red and down-regulated in green for each term. (**b**) A total of 860 DEGs were assigned to 85 KEGG pathways. The number of DEGs belonging to each category are provided.

A majority of up-regulated DEGs belonged to ‘protein synthesis and degradation’, ‘photosynthesis’ and ‘carbohydrate metabolic process’, while most of down-regulated DEGs were related to ‘defense response’ and ‘DNA-dependent regulation of transcription’ in the biological process category (Fig. 2a). Most of genes categorized in molecular function were involved in ‘catalytic activity’ and ‘binding activity’ (Fig. 2a, Supplementary Table S6). The top categories in cellular components included ‘integral to membrane’, ‘nucleus’ and ‘ribosome’ (Fig. 2a, Supplementary Table S6). A total of 860 DEGs from leaves of elevated CO_2_ grown *Jatropha* were assigned to 85 KEGG pathways (Fig. 2b). The most abundant KEGG pathways in our analysis were ‘ribosome’ (15%), ‘photosynthesis’ (11%) and ‘plant hormone signal transduction’ (11%) (Fig. 2b).

#### Photosynthesis and carbohydrate metabolism

Elevated CO_2_ conditions significantly induced the up-regulation of transcripts putatively encoding members of multi subunit complexes participating in light-dependent reactions. These include light harvesting chlorophyll a-b binding proteins, PSII protein complex components [D1 protein (PsbA), PS (II) CP47 chlorophyll apoprotein (PsbB), PS (II) CP43 chlorophyll apoprotein (PsbC), oxygen evolving complex protein (PsbP) and oxygen evolving enhancer protein 3-1], and PSI protein complex [PSI P700 chlorophyll a apoprotein A1 (PsaA)] (Table 3, Supplementary Table S6). Similarly, there was significant up-regulation of transcripts encoding NDH-1 plastoquinone reductase subunits B, K and T, ferredoxin-NADP^+^ reductase (FNR) and F-type H^+^-transporting ATPase subunit beta (FTA), which play an important role in photosynthetic electron transport and ATP synthesis (Table 3, Supplementary Table S6). The DGE analysis showed up-regulation in transcript levels of regulatory enzymes of carbon reduction cycle and pentose phosphate pathway including rubisco, both large (RL) and small subunit (RS), sedoheptulose-1,7-bisphosphatase (SB), phosphoribulokinase (PRK), transketolase (TKL), glucose-6-phosphate dehydrogenase (G6PD), ribose-5-phosphate isomerase (RI), chloroplastic triose phosphate isomerase (TPI) and chloroplastic NADP dependent glyceraldehyde-3-phosphate dehydrogenase (GAPDH) in elevated CO_2_ grown plants (Table 3, Supplementary Table S6

**Table 3:** Differentially regulated unigenes [log_2_fold change ≤−1.00 or ≥1.00 (elevated versus ambient)] associated with photosynthesis and carbohydrate metabolism in *Jatropha* after one year of growth under elevated CO_2_ identified in the RNA-seq analysis.

Pathway	Name	Number of unigenes	Gene ID	Regulation	Fold change
Photosynthesis: Light-dependent reactions	Chlorophyll a-b binding proteins	2	105637992	Up	~1.7
	D1 protein (PsbA)	1	7564824	Up	~2.6
	PS (II) CP47 chlorophyll apoprotein (PsbB)	1	7564760	Up	~1.4
	PS (II) CP43 chlorophyll apoprotein (PsbC)	1	7564849	Up	~1.7
	Oxygen evolving complex protein (PsbP)	1	105641916	Up	~1.6
	Oxygen evolving enhancer protein 3-1	5	105632870	Up	~1.7
	PSI P700 chlorophyll a apoprotein A1 (PsaA)	1	7564856	Up	~2.1
	NDH-1 plastoquinone reductase subunits (subunit B)	1	7564781	Up	~2.2
	NDH-1 plastoquinone reductase subunits (subunit K)	1	7564864	Up	~2
	NAD(P)H-quinone oxidoreductase subunit T, chloroplastic	3	105636994	Up	~1.6
	Ferredoxin-NADP^+^ reductase (petH)	7	105635331	Up	~1.6
			105629056		
	F-type H^+^-transporting ATPase subunit beta	1	7564869	Up	~1.6
Carbon reduction cycle & Pentose phosphate pathway	Rubisco large subunit	1	7564870	Up	~2.3
	Rubisco small subunit	2	105642030	Up	~1.5
	Sedoheptulose-1,7-bisphosphatase	3	105646676	Up	~1.5
	Phosphoribulokinase	2	105631767	Up	~1.8
	Transketolase	2	105643137	Up	~1.5
	Glucose-6-phosphate dehydrogenase	1	105641505	Up	~1.1
	NADP dependent glyceraldehyde-3-phosphate dehydrogenase	2	105630249	Up	~1.5
	Triose phosphate isomerase, chloroplastic	1	105643783	Up	~1.5
	Ribose-5-phosphate isomerase	2	105647678	Up	~1.7
			105636799		
Starch and sucrose metabolism	Granule bound starch synthase	8	105639084	Up	~1.7
	Phosphoglucomutase	3	105634080	Up	~2.1
	Sucrose phosphate synthase 1	2	105637611	Up	~2.4
	Sucrose phosphate synthase 3	1	105633156	Down	~1.9
	Fructose 1,6-bisphosphatase, cytosolic	3	105629115	Up	~1.6
Glycolysis	Hexokinase 1	4	105631535	Down	~1.1
	Hexokinase 3	1	105648314	Up	~1.6
	Phosphofructokinase	2	105632412	Up	~1.4
	Aldolase, cytosolic	5	105639139	Up	~1.2
			105643837		
	Triose phosphate isomerase, cytosolic	7	105647635	Up	~1.5
Others	Chloroplastic triose phosphate/phosphate translocator	11	105649590	Up	~1.5
	Chloroplastic glucose 6-phosphate/phosphate translocator	2	105645263	Down	~1.8
	Rubisco activase	5	105647331	Up	~1.6
	Carbonic anhydrase	4	105641553	Up	~2.1
			105640386		
Chlorophyll metabolism	Uroporphyrinogen decarboxylase	4	105637012	Up	~1.6
	Magnesium-protoporphyrin O-methyltransferase	1	105648719	Up	~1.5
	Chlorophyllase-1	3	105649943	Up	~1.6
Cell wall metabolism and remodelling	Cellulose synthase-like protein D1	1	105650364	Down	~2.9
	Xyloglucan glycosyltransferase 4	3	105642017	Down	~2.4
	Xyloglucan glycosyltransferase 12	5	105630800	Down	~2.1
	Endoglucanase 16	1	105638367	Down	~5.1
	Endoglucanase 17	1	105642560	Up	~1.6
	Pectin acetylesterase	1	105643391	Down	~1.8
	Pectin methylesterases	10	105642557	Down	~3.9
			105638004		
			105638008		
			105647823		
			105642556		
Cell wall metabolism and remodelling	Pectin methylesterases	4	105629307	Up	~1.9
			105632266		
	Polygalacturonases	1	105644635	Up	~3.2
	Polygalacturonases	5	105648836	Down	~2.3
			105629564		
	Expansin-A11	2	105635297	Up	~3.1
	β-expansin 3	6	105643364	Up	~1.6
			105643365		
	XTH1	1	105630427	Up	~1.8
	XTH7	4	105639898	Up	~1.6
	XTH22	1	105635614	Down	~1.5
	XTH23	18	105635617	Down	~3.5
			105635618		
			105629273		
			105629275		
			105635620		
	XTH30	1	105640962	Down	~3.2

The gene IDs were assigned according to Jatropha genome version JatCur_1.0 (http://www.ncbi.nlm.nih.gov/genome/jatrophacurcas) with a blast score ≥80.

).

Further, the expression of DEGs putatively encoding granule bound starch synthase (GBSS), hexokinase 3 (HXK3), phosphofructokinase (PFK), cytosolic aldolase, cytosolic TPI, sucrose phosphate synthase 1 (SPS1), fructose 1,6-bisphosphatase (FB), and phosphoglucomutase (PGM) was found to be up-regulated (Table 3, Supplementary Table S6). Interestingly, we found unigenes putatively encoding hexokinase 1 (HXK1) and sucrose phosphate synthase 3 (SPS3) to be down-regulated (Table 3, Supplementary Table S6). Also, significant up-regulation in transcripts encoding for chloroplastic triose phosphate/phosphate translocator (TPT) was noticed while down-regulation for chloroplastic glucose 6-phosphate/phosphate translocator (GPT) indicating increased exchange of triose phosphates between chloroplast and cytosol (Table 3, Supplementary Table S6). Significant up-regulation was also recorded for sequences putatively encoding for carbonic anhydrase (CA) and rubisco activase (RA) (Table 3, Supplementary Table S6).

#### Chlorophyll metabolism

The chlorophyll synthase gene showed no significant expression change under CO_2_ enriched conditions in *Jatropha*. Uroporphyrinogen decarboxylase, which catalyzes the first committed step of chlorophyll biosynthesis, and magnesium-protoporphyrin O-methyltransferase encoding transcripts were the only two members of chlorophyll biosynthesis which were significantly up-regulated (Table 3, Supplementary Table S6). Also, chlorophyllase-1, which participates in chlorophyll breakdown was found to be up-regulated in elevated CO_2_ treated plants (Table 3, Supplementary Table S6).

#### Cell wall metabolism and remodelling

There was significant differential regulation recorded for transcripts associated with cell wall metabolism and remodelling (Table 3, Supplementary Table S6). Genes involved in hemicellulose metabolism, cell wall loosening and expansion were differentially regulated in *Jatropha* under elevated CO_2_.

#### Transcription factors

We identified representations from known TF families reported for plants and also putative uncharacterized TFs in the assembled unigenes from ambient and elevated CO_2_-grown *Jatropha* plants (Supplementary Fig. S8). The differentially expressed family of TFs identified were MYB (up – 3; down – 14), bHLH (up – 8; down – 10), GATA (up – 5; down – 1), NAC (up – 3; down – 12), nuclear (up – 2; down – 11), MADS (up – 3; down – 7), WRKY (down – 20), AP2/ERF (up – 4; down – 23), zinc finger (up – 2; down – 14) and others (up – 27; down – 50) (Supplementary Fig. S9; Supplementary Table S7).

### Temporal expression pattern of key regulatory genes associated with photosynthesis

**Figure 3:**
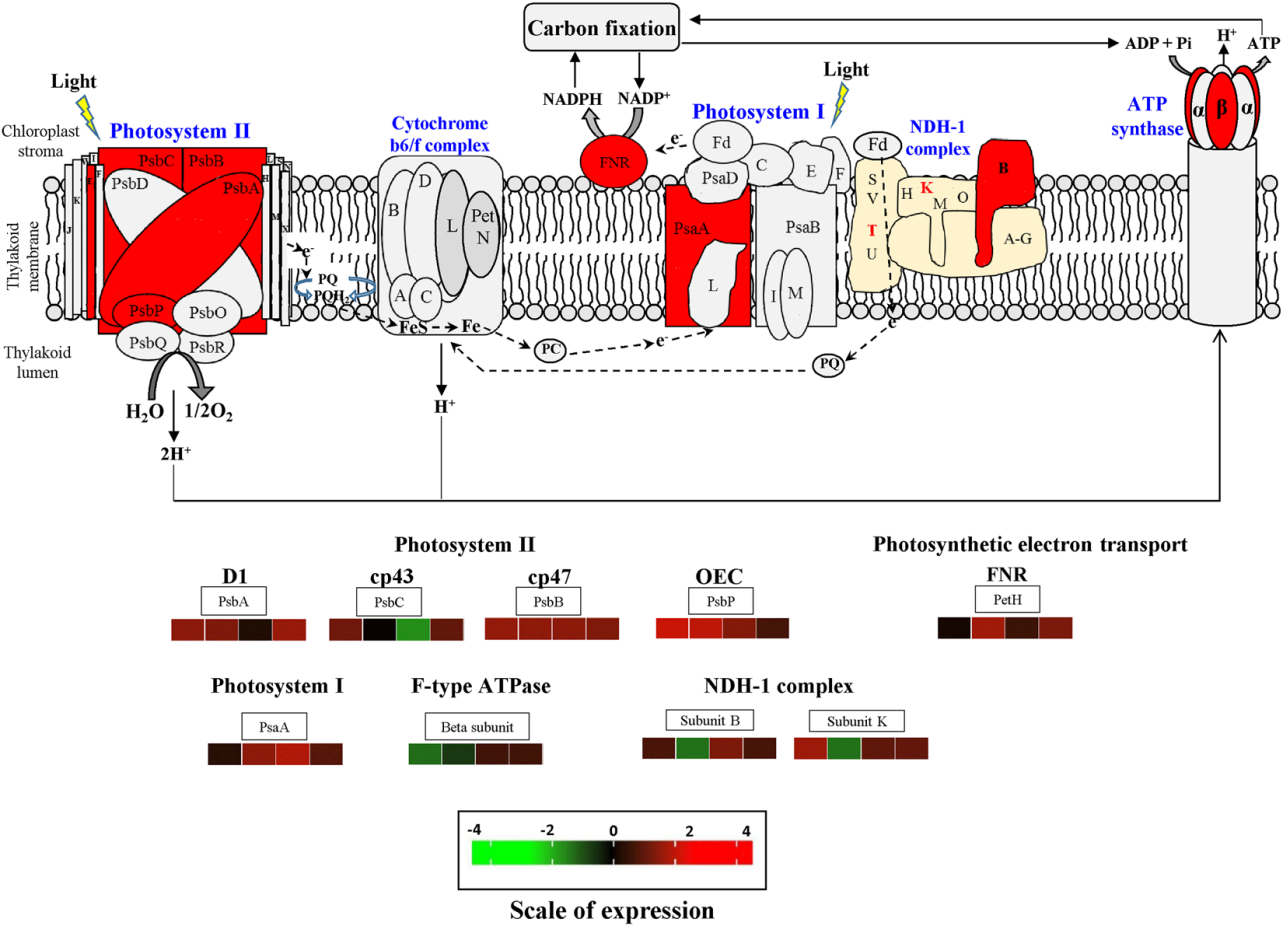
A comprehensive photosynthesis pathway depicting differentially expressed unigenes in multi-subunit complexes (photosystem I and II, NADH dehydrogenase and ATP synthase) participating in light-dependent reactions. The different subunits differentially regulated of each components (identified by differential gene expression analysis) are indicated in red and their expression patterns at different time points (90, 180, 270 and 360 DAT) in elevated CO_2_ grown plants with respect to ambient plants are represented. Different colour shades represent level of expression with red representing up-regulation while green as down-regulation. The level of regulation was determined based on log_2_ fold change. The four different boxes beneath each differentially regulated gene represents the four time points starting from 90 DAT to 360 DAT (direction: left to right). [Abbreviations for different subunits: PSII reaction centre core protein, D1 protein (PsbA); PS (II) CP47 chlorophyll apoprotein (PsbB); PS (II) CP43 chlorophyll apoprotein (PsbC); subunit of oxygen evolving complex protein (PsbP); PSI P700 chlorophyll a apoprotein A1 (PsaA); NADH dehydrogenase or NDH-1 plastoquinone reductase subunits B and K; petH; ferredoxin-NADP^+^ reductase (FNR); F-type H^+^-transporting ATPase subunit beta (FTA)].

To understand the expression trends of key regulatory genes of photosynthesis and associated metabolism for carbon conversion, we quantified the expression of thirty-five identified DEGs in leaves collected at four different time points [90, 180, 270 and 360 days after treatment (DAT)] during the course of growth of plants in elevated CO_2_ through qRT-PCR. The temporal expression of DEGs corresponding to members of multi subunit complexes associated with light-dependent reactions including subunits of PS II (PsbA, PsbB, PsbC and PsbP), PS I (PsaA), NADH dehydrogenase complex (B and K), FNR and ATP synthase complex (FTA) were found to be either up-regulated or no change at different time points of growth in elevated CO_2_ (Fig. 3, Supplementary Fig. S10). However, down-regulation was recorded for PsbC (270 DAT), subunits B and K (180 DAT) and FTA (90 DAT) (Fig. 3, Supplementary Fig. S10).

**Figure 4:**
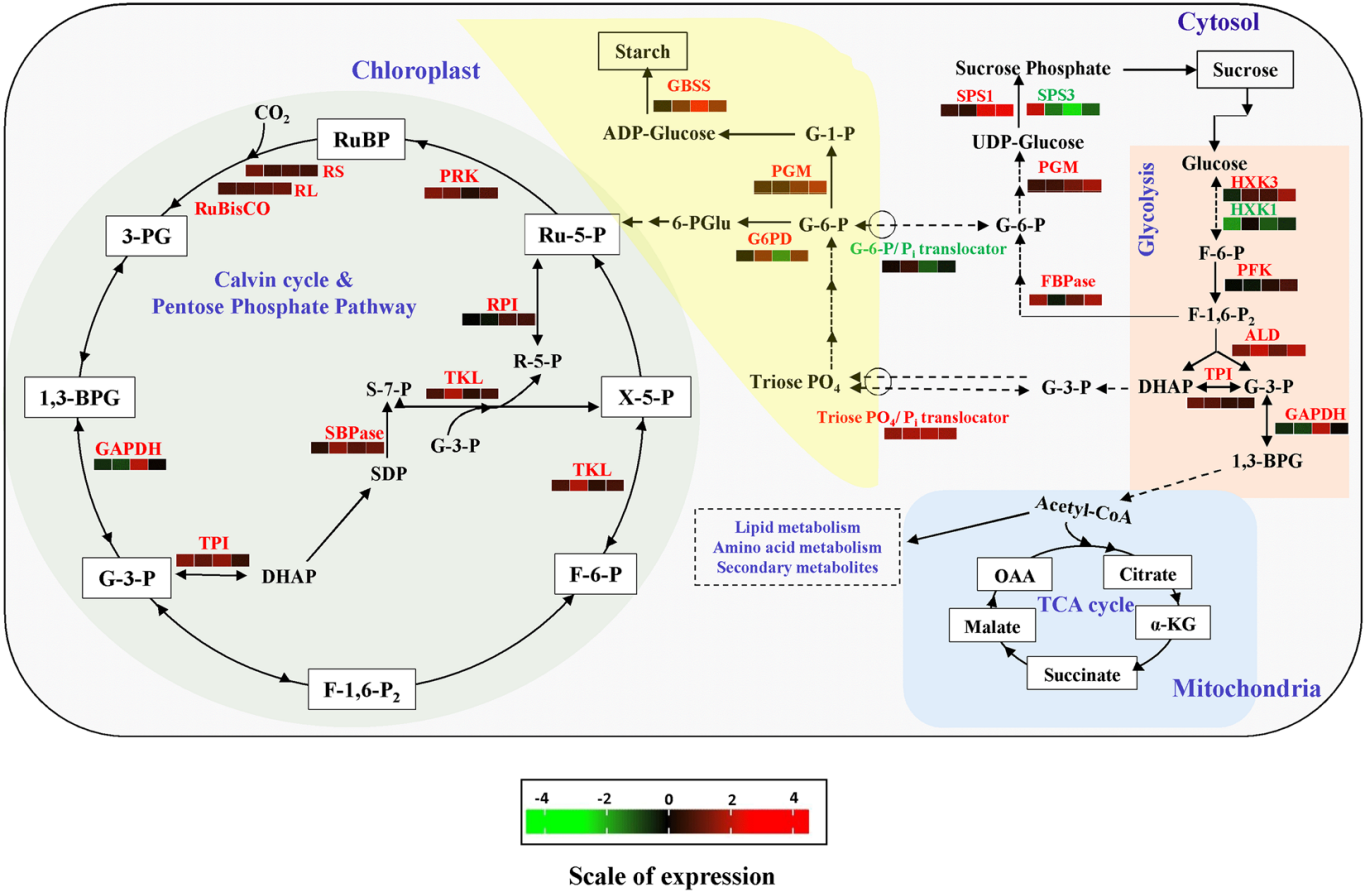
Schematic representation of temporal expression pattern of carbon metabolic hub involving Calvin-Benson cycle, pentose phosphate pathway, glycolysis, sucrose metabolism and starch biosynthesis. The expression levels of crucial regulatory genes (identified from transcriptome analysis) that may play critical roles in driving carbon conversion and exchange of intermediates between chloroplast and cytosol involving Calvin-Benson cycle, pentose phosphate pathway, glycolysis, sucrose metabolism and starch biosynthesis in elevated CO_2_ grown plants at different time points (90, 180, 270 and 360 DAT) with respect to ambient CO_2_ grown plants were represented as colored boxes. Different colour shades represent level of expression with red representing up-regulation while green as down-regulation. The level of regulation was determined based on log_2_fold change. The four different boxes beneath each differentially regulated gene represents the four time points starting from 90 DAT to 360 DAT (direction: left to right). [Abbreviations for metabolites and enzymes: RuBP - ribulose biphosphate; RuBisCO - ribulose-1,5-bisphosphate carboxylase/oxygenase; RS – Rubisco small subunit; RL – Rubisco large subunit; 3-PG – 3-phosphoglycerate; 1,3-BPG – 1,3-bisphosphoglycerate; GAPDH - glyceraldehyde 3-phosphate dehydrogenase; G-3-P - glyceraldehyde 3-phosphate; DHAP - dihydroxyacetone phosphate; TPI – triose phosphate isomerase; ALD – aldolase; F-1,6-P_2_ – fructose 1,6-bisphosphate; SDP – sedoheptulose 1,7-bisphosphate; SBPase – sedoheptulose 1,7-bisphosphatase; S-7-P - sedoheptulose 7-phosphate; TKL – transketolase; FBPase – fructose 1,6-bisphosphatase; F-6-P – fructose 6-phosphate; X-5-P – xylulose 5-phosphate; R-5-P – ribose 5-phosphate; RPI - ribose-5-phosphate isomerase; Ru-5-P – ribulose 5-phosphate; PRK – phosphoribulokinase; 6-PGlu – 6-phosphogluconolactone; G6PD – glucose-6-phosphate dehydrogenase; Triose PO_4_ – triose phosphates; G-6-P – glucose 6-phosphate; PGM – phosphoglucomutase; G-1-P – glucose 1-phosphate; SS – starch synthase; GBSS – granule bound starch synthase; G-6-P/Pi translocator – glucose 6-phosphate/phosphate translocator; F-6-P – fructose 6-phosphate; SPS1/SPS3 – sucrose phosphate synthase 1/sucrose phosphate synthase 3; HXK1/HXK3 – hexokinase 1/hexokinase 3; PFK – phosphofructokinase; OAA - oxaloacetic acid; α-KG – alpha-ketoglutarate].

DEGs pertaining to carbon conversion also showed varied temporal expression trends in the leaves of elevated CO_2_ grown *Jatropha* (Fig. 4, Supplementary Fig. S10). RL, RS, RA, PRK, TKL, chloroplastic TPI, SB, GAPDH, RI, CA, G6PD, GBSS, PGM, FB, PFK, cytosolic aldolase and cytosolic TPI was either significantly up-regulated or showed no change in expression at different time points of growth (Fig. 4, Supplementary Fig. S10). Only G6PD, a key regulatory enzyme of pentose phosphate pathway, was down-regulated at 270 DAT. A very interesting pattern of differential regulation was recorded for isoforms of HXK and SPS at the four time points. HXK1 was significantly down-regulated but HXK3 was up-regulated at different time points (Fig. 4, Supplementary Fig. S10). Similarly, SPS1 showed up-regulation while SPS3 was differentially regulated (Fig. 4, Supplementary Fig. S10). TPT showed significant up-regulation at all the four time points (Fig. 4, Supplementary Fig. S10). However, GPT showed significant down-regulation at 270 DAT with no significant change in expression recorded for other time points (Fig. 4, Supplementary Fig. S10).

Similarly, nitrogen metabolism associated gene nitrate reductase (NR) and glutamine synthetase (GS) was also significantly induced under elevated CO_2_ (Supplementary Fig. S10). However, glutamate dehydrogenase (GD) showed basal level of expression at all the four time points of growth in *Jatropha* under elevated CO_2_ (Supplementary Fig. S10).

### Enzyme activities of certain key regulatory enzymes associated with photosynthetic carbon reduction cycle

**Figure 5:**
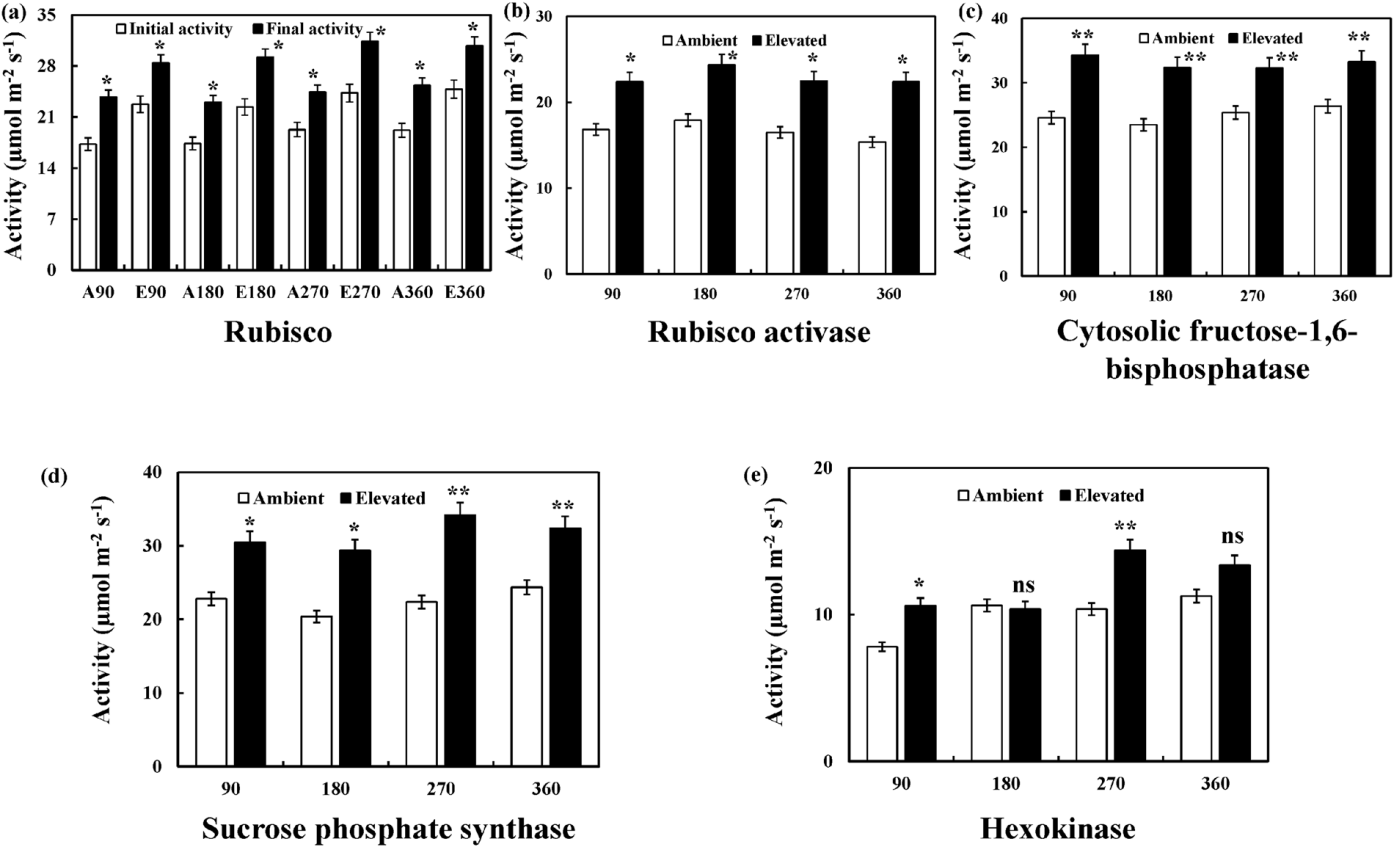
Enzyme activities of key regulatory enzymes associated with photosynthetic carbon reduction cycle in *Jatropha* leaves at four different time points (90, 180, 270 and 360 DAT). The enzyme activities of (**a**) rubisco, (**b**) rubisco activase, (**c**) cytosolic fructose-1,6-bisphosphatase, (**d**) sucrose phosphate synthase and (**e**) hexokinase at all the four time points in the leaves of ambient (white bar) and elevated (black bar) CO_2_ grown *Jatropha* plants. Values are expressed as mean ± SD. [Note – ns, not significant; *P < 0.05; **P < 0.01].

The enzyme activities of key regulatory enzymes of photosynthetic carbon reduction cycle positively correlated with the expression levels of the corresponding transcripts at the four time points. Both the initial and final activities of rubisco was recorded to be ~25% significantly higher (*P* < 0.05) in the leaves of elevated CO_2_ grown *Jatropha* at all four time points in comparison to ambient grown plants (Fig. 5a). Similarly, RA, cytosolic FB and SPS demonstrated significantly higher (*P* < 0.05; *P* < 0.01) activities at all four time points in elevated conditions (Fig. 5b–d). The activity of HXK at 90 and 270 DAT was recorded to be significantly (*P* < 0.05) higher by ~40% in elevated conditions (Fig. 5e). However, there was no significant variation at 180 and 360 DAT, the reproductive growth phase of both seasons, recorded in the activity of HXK in elevated CO_2_ conditions (Fig. 5e).

## Discussion

A better understanding of molecular responses under elevated CO_2_ can aid in modifying and managing plants to take maximum advantage of globally increasing CO_2_ which is also emphasized in earlier reports^23, 24^. In the present study, we combined transcriptomics with morphophysiology to investigate further the molecular mechanisms underlying the improved photosynthesis under elevated CO_2_ in *Jatropha*. The library preparation in our study was significantly good as demonstrated by the assembly statistics in which N50 value, average contig length, total number of contigs, longest contig length and number of assembled unigenes were higher in both sample A and E libraries in comparison to similar studies on *Jatropha* transcriptome under different conditions^21, 25^.

A yearlong growth spread across two growth cycles allowed us to assess the consistency of response of *Jatropha* in CO_2_ enriched environment for two different seasons. *Jatropha* exhibited a net increase in photosynthetic activity, fast growth, increased biomass production and was not sink-limited at either growth seasons under elevated CO_2_. The growth and development of *Jatropha* shows distinct pattern depending on the environmental conditions^26^. A very interesting observation recorded was significant reproductive yield was recorded for *Jatropha* at 180 DAT under elevated CO_2_ unlike ambient conditions which showed normal vegetative behaviour with very little or no fruits suggesting elevated CO_2_ ameliorated the negative effects of temperature on the fruit formation and development^26, 27^. This clearly indicates the efficiency of *Jatropha* to positively maintain better resource utilization towards sink development in the form of biomass and fruit yield under elevated CO_2_ environment irrespective of season. These morphophysiological alterations involve a transcriptomic reprogramming which includes DEG belonging to diverse GO and KAAS terms as evident in our annotation results. Accordingly, the expression profiles were tailored in *Jatropha* to modulate appropriate metabolic pathways in a coordinated manner to produce a coherent response towards sustaining enhanced photosynthesis in the elevated CO_2_ environment.

The physiological results demonstrate that increased atmospheric CO_2_ stimulated light-saturated photosynthesis in *Jatropha* through increased absorption of photosynthetic photon flux density to be used for photochemistry and associated light reactions. These physiological findings were further corroborated by RNA-Seq and qRT-PCR assisted temporal expression analysis. Significant up-regulation in transcripts pertaining to PsbA, PsbB and PsbC in PSII as well as PsaA subunit of PSI under elevated CO_2_ at different time points suggest an efficient energy transfer in the form of electrons from antenna complexes to photosystems reaction core for meeting the demands of energy requirement in the carbon reduction cycle^28^. Increased *F*_*v*_ */F*_0_ implies efficient water splitting complex on the donor side of the PS II, resulting improved photosynthetic electron transport capacity. This may be correlated with the up-regulation in transcript of PsbP subunit of oxygen evolving complex suggesting enhanced photooxidation of water and electron transport rates in *Jatropha* under elevated CO_2_. However, down-regulation and no change in expression recorded for these genes at some time points indicate possible maintenance of balance in utilizing the light energy for electron transfer at different stages of growth which might involve other PSII and PSI subunits not identified as differentially regulated in this study^29^. FNR demonstrated interesting expression trend with up-regulation during reproductive development and maturation period (180 and 360 DAT) of *Jatropha* under elevated CO_2_ suggesting an enhanced electron transport rates in coordination with other subunits to facilitate increase in reducing equivalent production to be utilised in carbon reduction cycle. However, no significant variation in expression during vegetative growth stage suggest possible involvement of other subunits associated with photosynthetic electron transport which has several rate-limiting steps, with FNR catalysing just one of them^30^. Similarly, differential regulation of both subunits of NDH complex, B and K suggests an optimum balance of electron flow around PSI and PSII in maintaining increased photosynthesis capacity of *Jatropha* under elevated CO_2_^31, 32^. There was no correlation in the DGE expression and qRT-PCR expression of FTA indicating probable involvement of other subunits in optimising sustained ATP synthesis to maintain enhanced photosynthesis in elevated CO_2_ grown *Jatropha* ^33^. Our data on photochemical and non-photochemical quenching clearly indicate that most of the absorbed light energy was efficiently used in qP rather than NPQ in elevated CO_2_-grown *Jatropha* plants. Our results also demonstrated no significant changes in the chlorophyll concentrations and chlorophyll a/b ratio in elevated CO_2_ conditions at all growth stages regardless of high photosynthetic rates which suggests efficient management of light absorption capacity and radiative energy balance of the photosystems to sustain high photosynthetic rates^34^. The up-regulation of transcripts associated with both chlorophyll biosynthesis and breakdown in the DGE analysis indicate a possible co-regulation to maintain the chlorophyll concentrations in *Jatropha* under elevated CO_2_ conditions. Altogether, elevated CO_2_ increased photosynthesis, photochemistry (overall efficiency of PSII performance) and linear electron flow in *Jatropha* suggesting PSII-adaptive improved growth and productivity performance in elevated CO_2_ environment.

The most important pathway for any plant is the photosynthetic carbon reduction (PCR) cycle which fixes the atmospheric CO_2_ into organic forms to be utilized for growth and development. Most evidently, the DGE and temporal expression analysis showed up-regulation in transcript levels of enzymes of PCR cycle including rubisco (both RL and RS), SB, and PRK in elevated CO_2_ grown *Jatropha* plants. The temporal expression pattern of rubisco (both subunits), SB and PRK, especially at 180 and 360 DAT which is the seed setting stage of *Jatropha* under elevated CO_2_, indicates enhanced production of carbon intermediates for carbon fixation which correlates with our physiological and biochemical findings of sustained enhanced photosynthetic rates, biomass accumulation, reproductive yields and rubisco activity^35^. The up-regulation of these transcripts possibly ensures enhanced supply of carbon skeletons to manage the demand from newly formed reproductive sink tissues. Further, GAPDH showed up-regulation only at 270 DAT suggesting enhanced photosynthesis and photochemistry of *Jatropha* is independent of GAPDH expression under elevated CO_2_. The upregulation of RA at 180 DAT suggests activation and maintenance of rubisco catalytic activity by promoting the removal of any tightly bound, inhibitory, sugar phosphates from the catalytic site of both the carbamylated and decarbamylated forms of rubisco under elevated CO_2_^36^. However, there was no variations in expression levels of RA at 90, 270 and 360 DAT indicating balanced ATP/ADP ratios under elevated CO_2_ to sustain enhanced photosynthesis in *Jatropha* under elevated CO_2_^37^. Further, the measured RA enzyme activity did not correlate with the transcript levels which may be attributed to the complexity of the relationship between RNA levels, protein levels, and physiological changes^38^. We also observed significant enrichment in transcripts of enzymes participating in pentose phosphate pathway (PPP), G6PD, TKL and chloroplastic TPI which generates NADPH and carbon intermediates to meet the demand for carbon skeletons for both vegetative and reproductive sinks in elevated CO_2_ grown *Jatropha* during both growth seasons^39^. Furthermore, the expression analysis of RPI, which maintains pentose and hexose carbon pools in equilibrium with one another as the fluxes through the major metabolic pathways change in response to external environmental conditions did not correlate with DGE analysis with only up-regulation recorded at 270 DAT suggest involvement of other players in the regulatory network in maintaining the pentose and hexose carbon ratio^40^. An increase in foliar carbohydrate levels, particularly starch, is a common observation in elevated CO_2_ environments which has been predicted to be responsible for photosynthetic acclimation in the absence of sink tissues^6^. Our biochemical results demonstrated increased starch levels in leaf which correlated with significant upregulation in transcripts encoding granule bound starch synthase (GBSS) in elevated CO_2_ conditions^41^. We have earlier proposed that *Jatropha* was able to escape photosynthetic downregulation in the later stages of growth due to availability of sufficient sinks like increased tertiary branches, flowers and fruits to sustain enhanced growth^22^. This implies efficient source-sink interaction and sustained photosynthetic potential of *Jatropha* under elevated [CO_2_]. Moreover, differential regulation of nitrogen metabolism associated gene, NR indicate improved nitrogen allocation and nitrogen use efficiency in the leaves of elevated CO_2_ grown *Jatropha*. However, GS and GD did not demonstrate any differential expression in *Jatropha* under elevated CO_2_ environment indicating nitrogen use is not regulated by these two genes at these four growth stages.

Our foliar biochemical results in this study showed increase in soluble sugar content in elevated CO_2_-grown *Jatropha* plants. Starch and sucrose metabolisms are finely co-regulated involving key intermediate metabolites at the cross-road of catabolic and anabolic pathways for the control of carbon flux in the plant cell. There was significant up-regulation of transcripts pertaining to sucrose metabolism and glycolytic pathway. The significant up-regulation of transcripts pertaining to PGM, cytosolic FB, cytosolic TPI and cytosolic ALD imply improved sucrose metabolism in the cytosol^35^. The enzyme activity of cytosolic FB complied with the temporal expression patterns as indicated in our results. The up-regulation of these transcripts also imply increased hexose sugar pools for various carbon skeletons to be provided to the developing sinks under elevated CO_2_. However, PFK showed up-regulation at only 360 DAT suggesting its non-regulatory role under elevated CO_2_ as it has been implicated in adaptation of plants in non-optimal conditions^42^. Interestingly, we found differential regulation in transcripts encoding isoforms of hexokinase and sucrose phosphate synthase. The differential regulation of both SPS and HXK isoforms may have played a role in exerting regulatory influence on sucrose biosynthesis and hexose sugar pools to accommodate enhanced photosynthesis in *Jatropha* grown in CO_2_ enriched atmosphere^43^. Also, this suggests the optimum balance in the catalytic and signaling function of HXK and also SPS, which may be one of the adaptive strategy during morning hours for sustained photosynthesis in elevated CO_2_-grown *Jatropha* ^44^. The enzyme activity of SPS1 was in agreement with the transcript expression at all stages of growth while the activity of HXK did not alter at the reproductive development stage suggesting regulatory effect of sucrose on hexose sugar pools^45^. The cytosolic glycolytic network may provide an essential metabolic flexibility that facilitates plant growth and development under elevated CO_2_. We recorded significant up-regulation of transcripts pertaining to chloroplastic TPT at all stages of growth under elevated CO_2_ indicating a coordinated metabolic interaction between cytosol and chloroplast for high levels of carbon skeletons to be utilized for sustained growth and development^46^. The downregulation of GPT suggests preferential exchange of triose phosphate intermediates between subcellular compartments during morning and may be one of the adaptive strategy for sustained photosynthesis in *Jatropha*.

In conclusion, the present study highlights the importance of differential expression of key regulatory genes of photosynthetic electron transport in chloroplasts and central carbohydrate metabolism in maintaining the improved photosynthetic capacity during long-term growth in *Jatropha* under elevated CO_2_.

## Methods

### Plant material and CO_2_ treatment

*Jatropha curcas* seeds (Variety: CG-20) were surface sterilized with 0.5% sodium hypochlorite and grown in polythene bags. After 10 days, three *Jatropha* seedlings were transplanted in three pits with a spacing of 2 × 2 m in octagonal-shaped open top chambers (OTCs). The establishment of plant growth, experimental setup, microclimatic growth conditions and CO_2_ treatment inside the OTCs were same as described in our previous study^22^. Two OTCs were used for elevated CO_2_ treatment (mean CO_2_ concentration −550 ppm) and two for ambient CO_2_ (mean CO_2_ concentration −395 ppm) in this study. Three *Jatropha* plants were grown and maintained in each OTC for one year. The plants were maintained as coppice plantations after completion of harvest of seeds which was done once every six months. The experiment started in the month of January and the first growth season ended in June and the second growth season was from July to December. The two growth seasons were chosen with respect to the growth behaviour of *Jatropha* as described earlier^26^. Morphological and physiological parameters like plant height, light response curves (*A/Q* curves) and chlorophyll a fluorescence were performed after 90, 180, 270 and 360 days of growth on both ambient and elevated CO_2_ grown plants as described previously^22, 47, 48^. Similarly, chlorophyll, soluble carbohydrates and starch content were measured on the same leaves used for physiological measurements^22^. Above ground biomass and reproductive yields were assessed at 180 and 360 days after coppicing the plants. These morphological, physiological and biochemical measurements were performed to analyse the consistency in response of *Jatropha* under elevated CO_2_ at different seasons. Further, upper canopy young green leaves (preferably 3^rd^ from top) used for physiological measurements were collected at the above mentioned four time points (90, 180, 270 and 360 days) from both ambient and elevated CO_2_ grown *Jatropha* plants during its one year of growth and stored at −80 °C until use. Three independent biological replicates of stored young green leaves of 360 days from each ambient and elevated grown plants were used for sequencing (each replicate from individual tree). The transcriptome generated at this time point was used for deciphering differential regulation of key regulatory genes associated with photosynthesis and carbohydrate metabolism at the four different time points and correlate their expression patterns with morphophysiological analysis. The four time points selected for morphophysiological, biochemical and molecular analyses reflect two distinct stages of *Jatropha* across two seasons. The 90 and 270 days are the stages when the vegetative growth rate is at maximum for both seasons. At 180 and 360 days for both seasons, both vegetative and reproductive growth simultaneously occur.

### RNA extraction, Illumina sequencing and quality control

Agilent plant RNA isolation kit (Agilent Technologies, USA) was used to isolate total RNA from leaf tissue of ambient (sample A) and elevated (sample E) CO_2_-grown *Jatropha* plants respectively. The concentration, intactness and purity of RNA were checked with Agilent 2100 Bioanalyzer (Agilent Technologies, USA). Samples having RNA integrity number (RIN) value greater than 8 were used for library preparation. Three biological replicates were sequenced from both ambient and elevated CO_2_-grown *Jatropha* plants. Paired end cDNA library preparation for ambient and elevated samples was performed by the genomics facility (Genotypic Technology Pvt. Ltd., Bangalore, India) following Illumina TruSeq RNA library protocol outlined in “TruSeq RNA Sample Preparation Guide” (Illumina Technologies, San Diego, CA). The prepared library was quantified using Nanodrop and validated for quality by running an aliquot on High Sensitivity Bioanalyzer Chip (Agilent Technologies, USA). Sequencing of constructed cDNA library was performed on Illumina HiSeq. 2000 sequencer on high output mode and RNA-Seq data were generated in Fastq format. Sequencing resulted in generation of 101 bp raw reads having attached adapter sequences in tissues from both sample A and E. These raw reads were subjected to filtering through the standard Illumina pipeline. The filtered Fastq files were further subjected for quality control using SeqQC 2.1^49^ and BBDuk (https://sourceforge.net/projects/bbmap/) to remove adapters, B-block, low quality bases towards 3′ ends and contaminant reads.

### Sequence assembly and analysis

Assembly was performed using the high quality reads after removing duplicate reads from libraries for both the samples using Velvet 1.2.10 and Oases 0.2.08 at different k-mer lengths^50, 51^. The high quality filtered reads were *de novo* assembled for transcript generation. Various k-mer assemblies were performed and the best hash length assembly was selected (Sample A: 49, Sample E: 53) considering various parameters like total number of transcripts generated, maximum transcript length, total transcript length and less number of N’s. Further, as the *Jatropha* genome is available, the reference based assembly was also performed by mapping the trimmed reads onto the *J. curcas* reference genome and exclusively to gene models using Rsubread package on the downloaded files from NCBI (NCBI GCA_000696525.1) [http://www.ncbi.nlm.nih.gov/genome/annotation_euk/Jatropha_curcas/100/]^17, 52^. After mapping, the mapped file in *.bam format were used to count mapped reads and to provide the genomic coordinates, GTF file was downloaded from NCBI site. The mapped reads were counted using featureCounts program within the Rsubread package^53^. The unaligned reads to the genome were also assembled. The clustering of assembled transcripts from both libraries to generate unigenes was performed using CD-HIT^54^. The length of the assembled unigenes for further study were selected as ≥200. Gene IDs were assigned to unigenes according to *Jatropha* genome version JatCur_1.0 (http://www.ncbi.nlm.nih.gov/genome/jatrophacurcas).

### *In silico* differential gene expression analysis

The differential gene expression (DGE) analysis in the sequenced cDNA library generated from leaves of both sample A and E was carried out using DESeq tool, considering sample A as control and sample E as treated from the unigenes generated^55^. The alignment of reads of both sample A and E was performed using Bowtie tool^56^. The read count profile for reads from both A and E was generated and DGE analysis carried out using shrinkage estimation for dispersions and fold changes to improve stability and interpretability of estimates. The relative expression levels of each annotated unigene was estimated separately for both samples and also as a joint estimate from both samples and presented as its mean expression level (at the base scale), the fold change from sample A to sample E and the logarithm (to basis 2) of the fold change. An expression matrix at the gene and transcript level was also generated in the form of FPKM values for trimmed reads aligned to reference genome. The differentially expressed genes (DEGs) were identified with log-fold (log_2_) expression change ≥1 or ≤−1 using a statistically significant P-value (*P* < 0.05) and FDR (FDR < 0.01).

### Functional annotation of transcript sequences

Transcript annotation was done by performing BLASTX analysis against the non-redundant protein database (N_r_) and Swiss-Prot^57, 58^. The hits with an E-value ≤ 1E-05 and blast score ≥80 were considered to be significant. GO (Gene Ontology) terms were assigned to impart a broad overview of their functions and categorized into biological process, molecular function and cellular component using in house Perl scripts. Also, KOG (Eukaryotic Orthologous Groups) was used to identify the transcript homologues from other organisms and thus assigning a probable function to transcripts. KAAS [KEGG (Kyoto Encyclopedia of Genes and Genomes) Automatic Annotation Server] was used for metabolic pathway analysis using *Arabidopsis thaliana* and *Oryza sativa* L. ssp. *japonica* as reference organisms to identify the enriched metabolic pathways in various gene sets^59^. The unigenes were classified into various transcription factors (TFs) using Transcription factor Family Data Base (TFDB)^60^.

### Quantitative PCR analysis

Validation of differential gene expression data was carried out using the qRT-PCR analysis at the four different time points. Gene specific primers were designed for certain key regulatory enzymes involved in photosynthesis, carbohydrate and nitrogen metabolism (Supplementary Table S8). The qRT-PCR was performed on Eppendorf thermal cycler using KAPA SYBR FAST qPCR Master Mix (2X) Universal (KAPA Bio systems, USA) as described previously^61^ on the leaves collected at different time points including the ones used for transcriptome. The relative expression was calculated using the formula, F = 2^−(∆Ct treated−∆Ct control)^ with 18SrRNA as housekeeping gene for normalization of data^62^. The fold change values were log transformed with base 2 so that ~1.00 fold was used to identify differentially expressed genes.

### Enzyme activity measurements

The activity measurements of certain key regulatory enzymes of photosynthesis and carbohydrate metabolism like rubisco, SPS, HXK, FB and RA were performed on leaves of both ambient and elevated CO_2_ grown plants as described previously^63–65^ at all four time points.

### Statistics

For qRT-PCR analysis, three independent biological replicates with three technical replicates of each biological replicates for both samples were used for analysis and the mean ± standard deviation (SD) values were calculated for each sample. The significance of the difference for all physiological (n = 6–15), biochemical (n = 6–15) and molecular were tested by using Analysis of Variance (ANOVA) and the comparisons were tested by Dunnett’s multiple comparison analysis. All statistical analysis was performed using SIGMA PLOT 11.0.

## Electronic supplementary material

Supplementary information

Supplementary Table S1

Supplementary Table S4

Supplementary Table S5

Supplementary Table S6

Supplementary Table S7
