# Supplementary material for: Molecular insights into photosynthesis and carbohydrate metabolism in Jatropha curcas grown under elevated CO2 using transcriptome sequencing and assembly
Source: Sci Rep. 2017 Sep 11;7:11066. doi: 10.1038/s41598-017-11312-y (PMC5593950; doi:10.1038/s41598-017-11312-y)
Supplement: Supplementary file 1 — Supplementary information [file 41598_2017_11312_MOESM1_ESM.pdf]

Supplementary Information

Molecular insights into photosynthesis and carbohydrate metabolism in *Jatropha curcas* grown under elevated CO_2 using transcriptome sequencing and assembly

Sumit Kumar, Rachapudi Venkata Sreeharsha, Shalini Mudalkar, Prasad M Sarashetti and Attipalli Ramachandra Reddy

Supplementary Figure S1

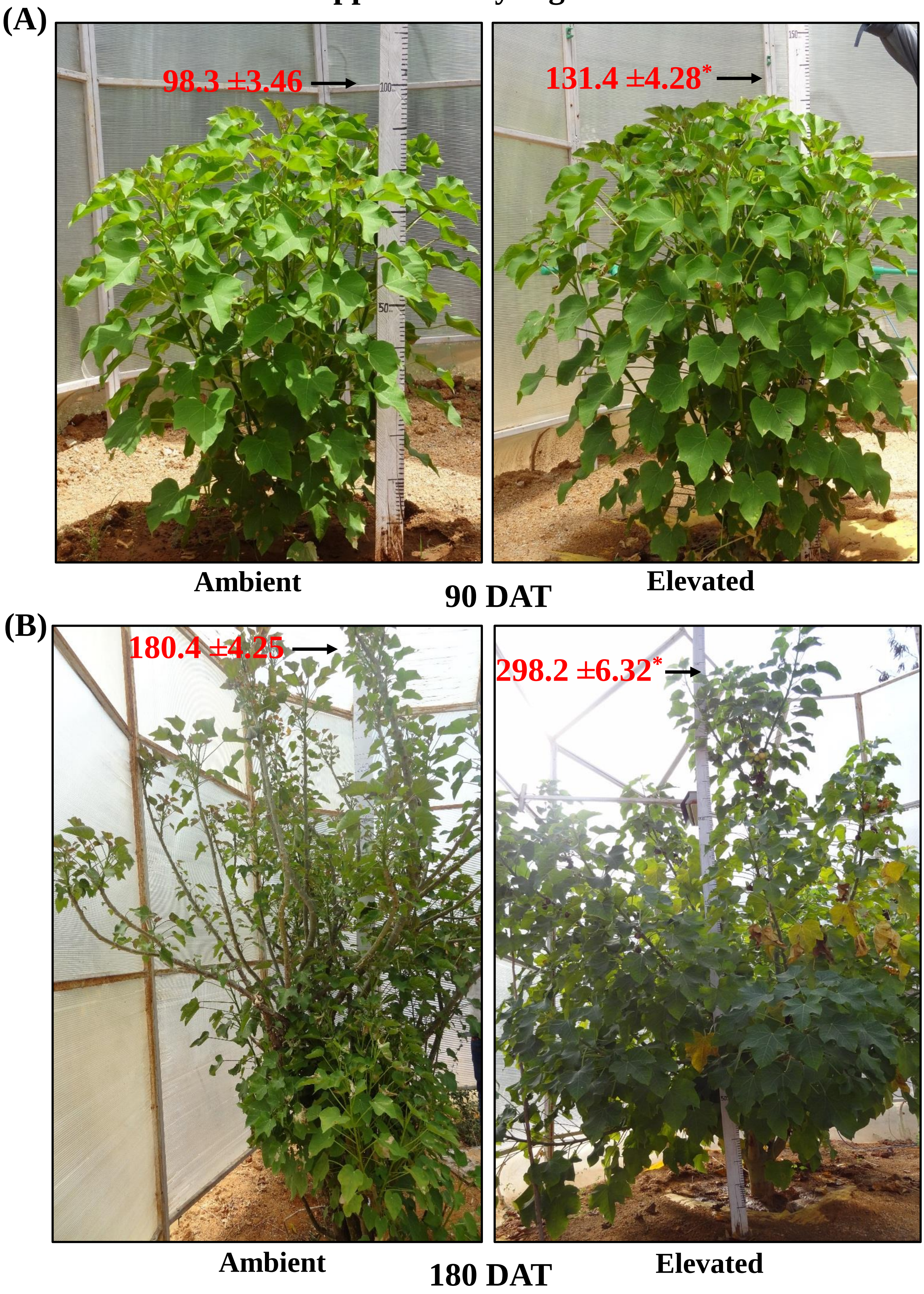

Supplementary Figure S1. Plant height and morphology of *Jatropha* during first growth season at 90 and 180 DAT under ambient and elevated conditions

Supplementary Figure S2

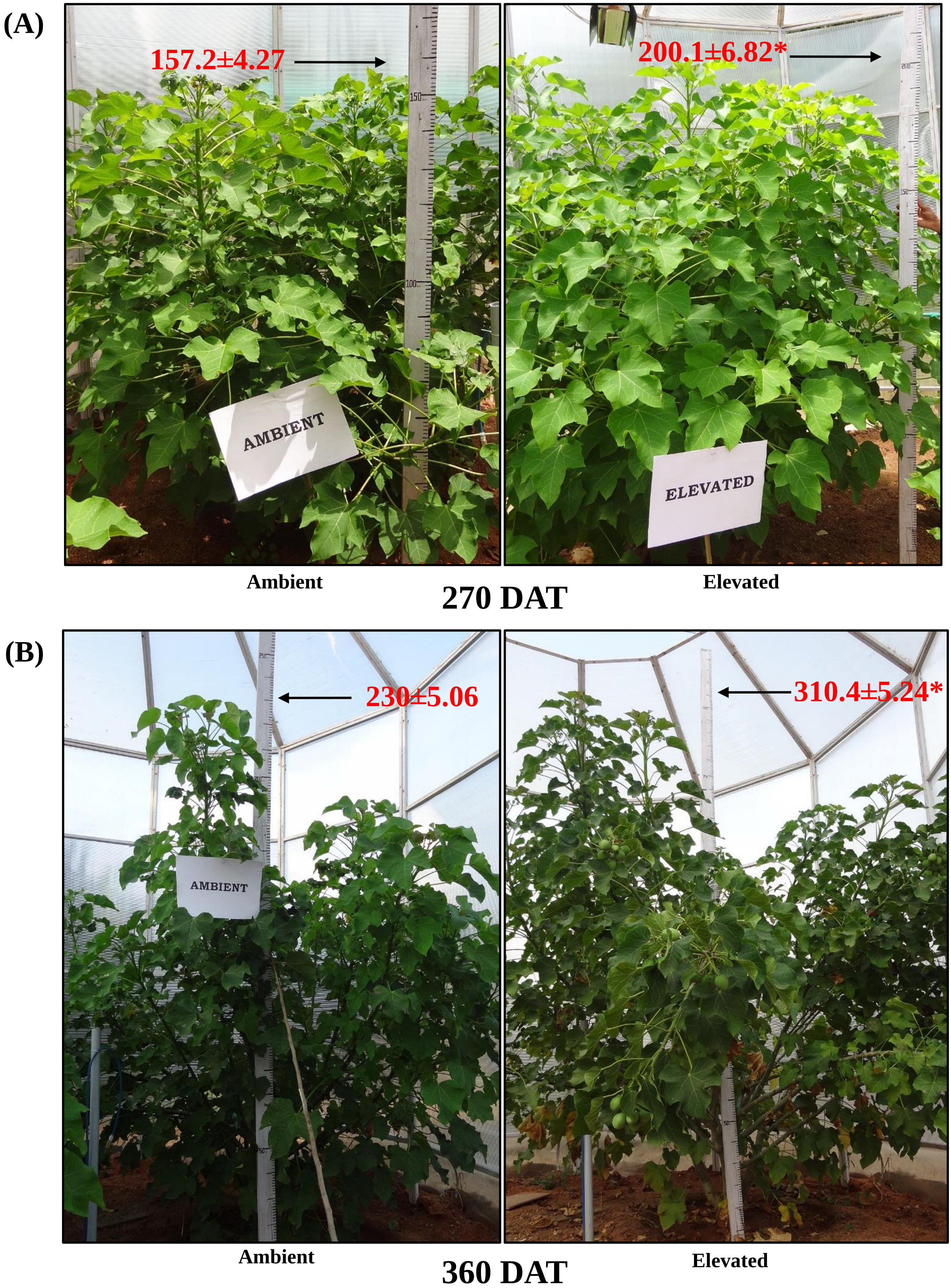

Supplementary Figure S2. Plant height and morphology of *Jatropha* during second growth season at 270 and 360 DAT under ambient and elevated conditions

Supplementary Figure S3

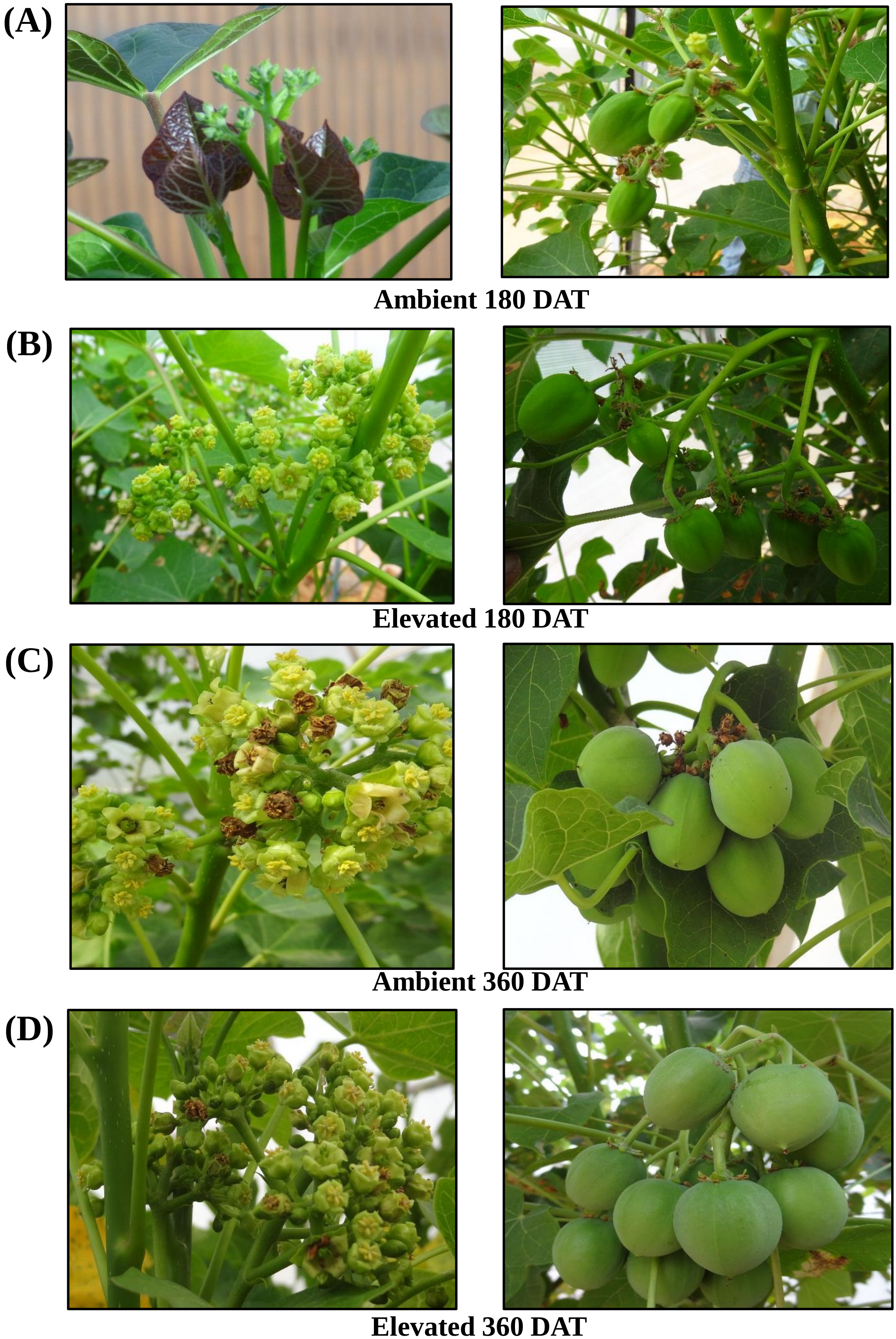

Supplementary Fig. S3. Comparative flower and fruit morphology of *Jatropha* grown under ambient and elevated CO_2 conditions at the end of two growth seasons (180 and 360 DAT). The flowers in ambient conditions during first growth season showed reduced growth due to high temperature which also showed its aftereffect in poor development and number of fruits. However, during second growth season (optimum temperature) they showed better growth and morphology which was translated in the normal fruit development but less in number when compared to elevated conditions. The elevated grown plants showed normal flower and fruit development during both seasons with larger numbers recorded in comparison to ambient plants.

Supplementary Figure S4

(a) Ambient

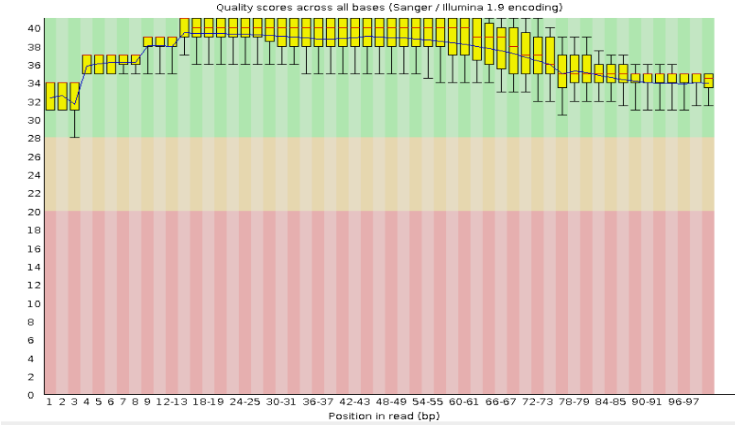

Paired-end 1

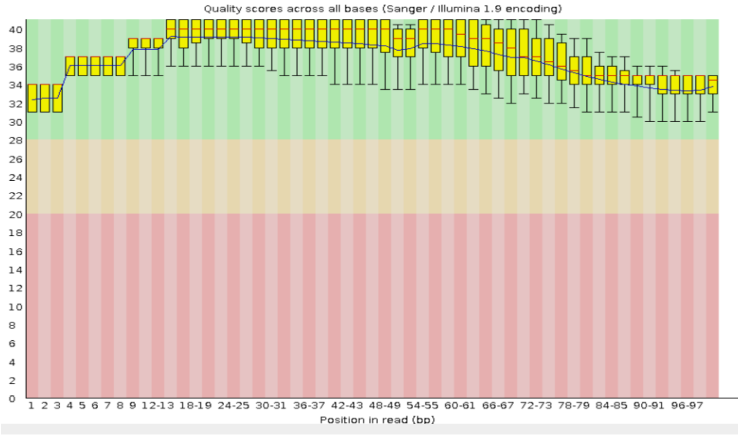

Paired-end 2

(b) Elevated

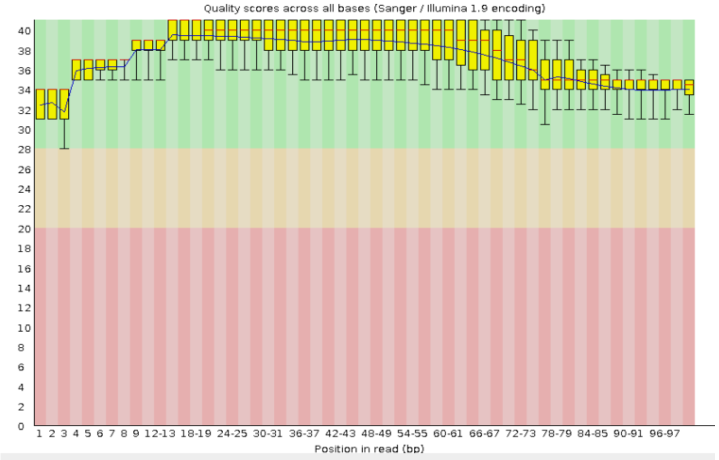

Paired-end 1

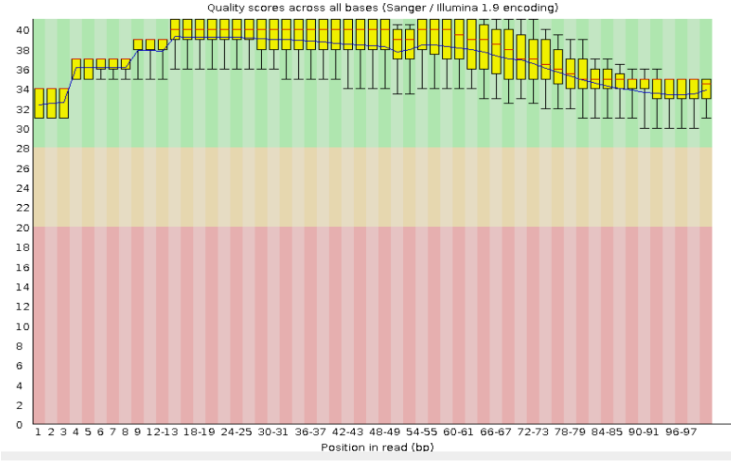

Paired-end 2

Supplementary Fig. S4. Average Phred quality scores of each base position for filtered reads for both the paired-end reads in (a) ambient and (b) elevated conditions

Supplementary Figure S5

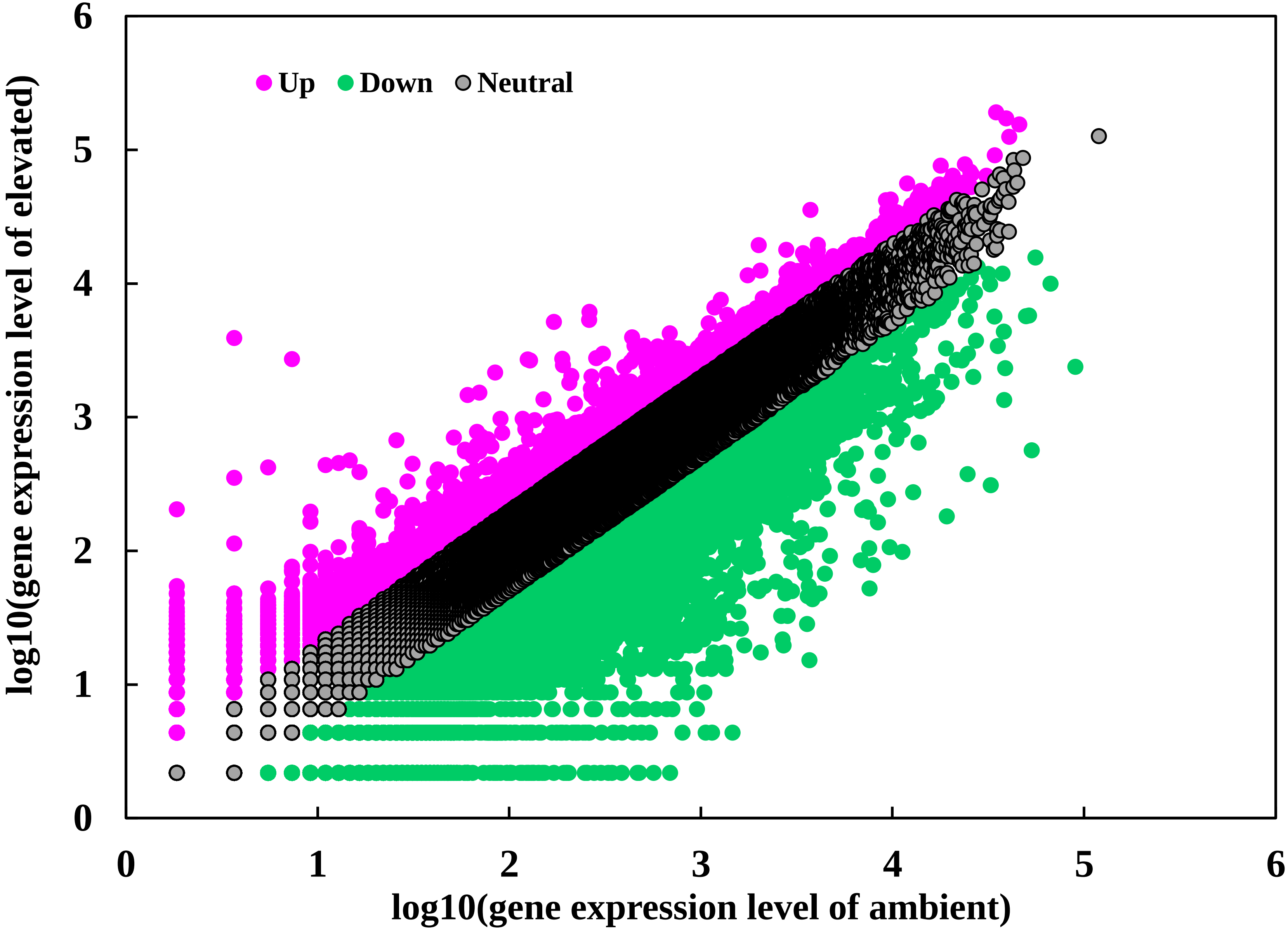

Supplementary Fig. S5. Differentially expressed genes in the leaves of elevated CO_2 compared with those in the ambient conditions. The significantly up-regulated genes by elevated CO_2 treatment were marked in magenta pink while the significantly down-regulated genes were marked in green with the threshold of (log_2Ratio) ≥ 1.

Supplementary Figure S6

(a)

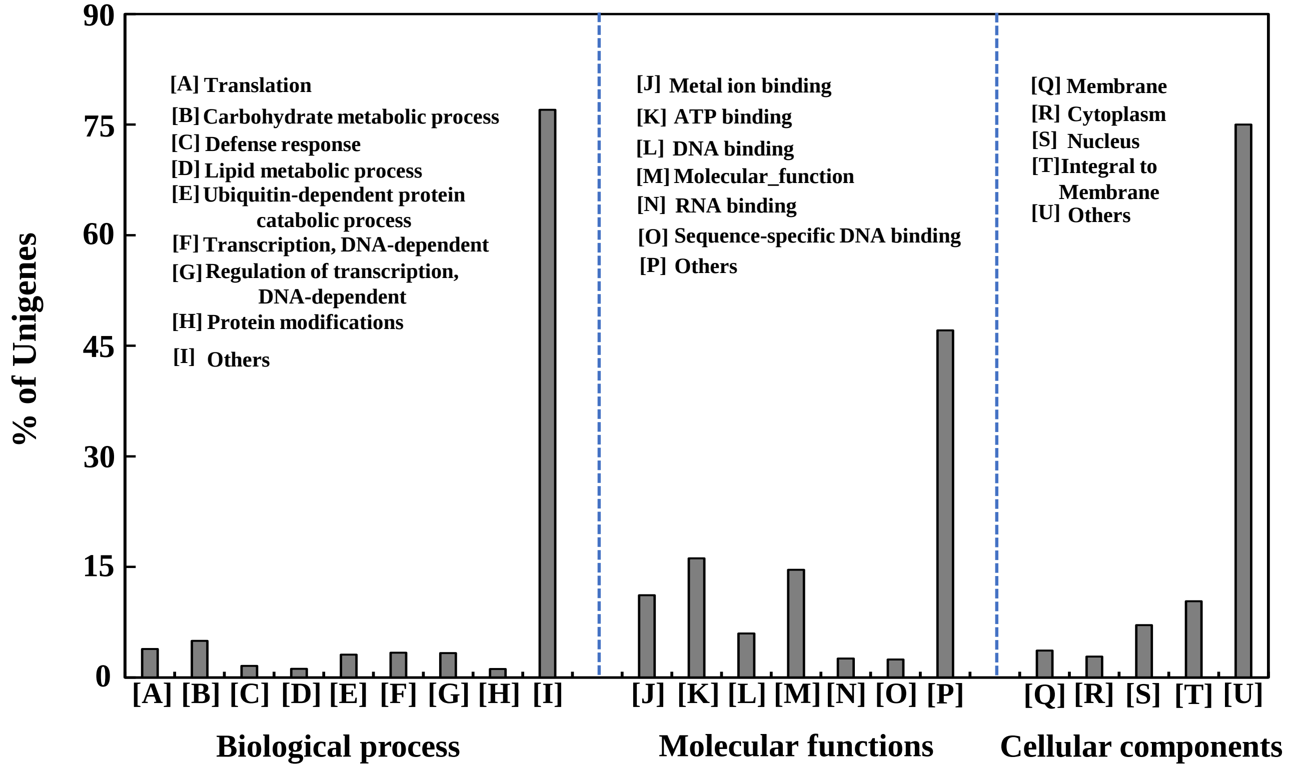

(b)

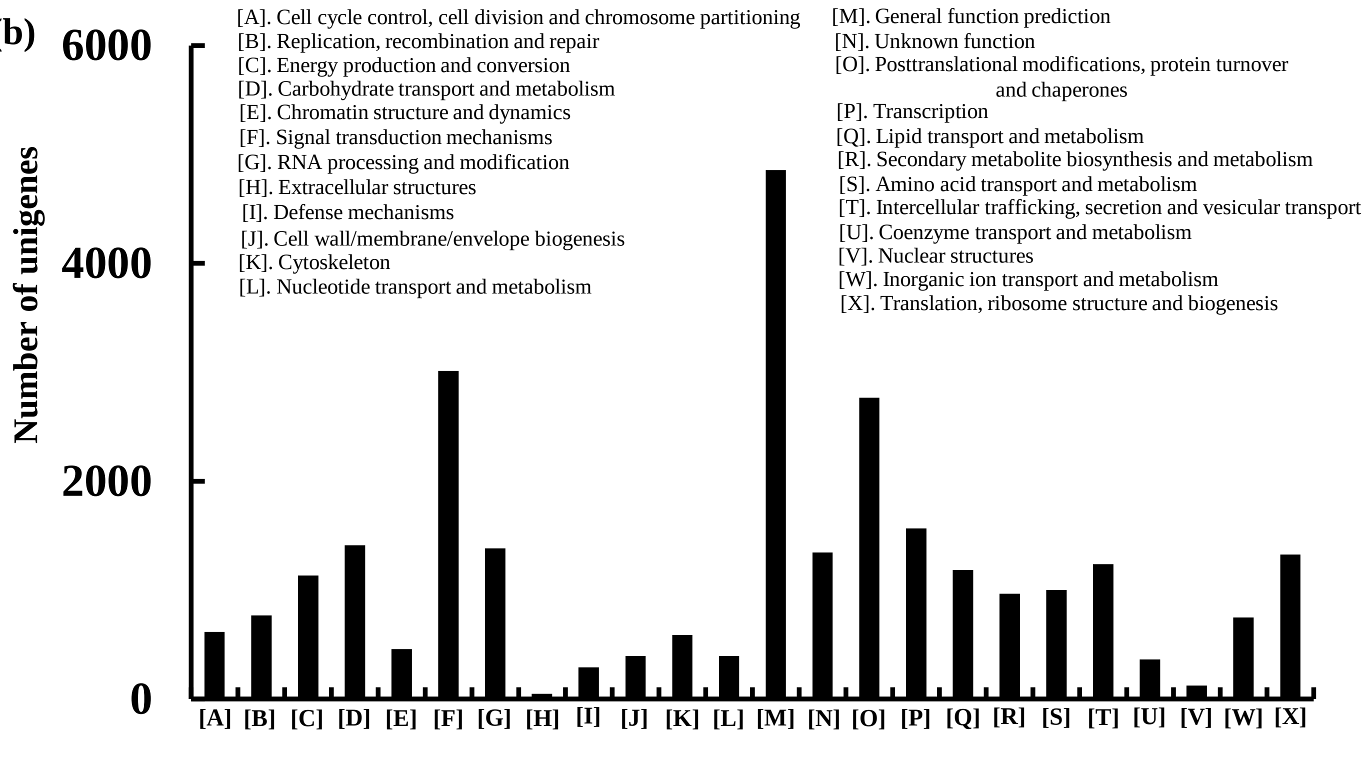

Supplementary Fig. S6. (a) GO annotations for *Jatropha* leaf unigenes. (b) Distribution of unigenes according to KOG database.

Supplementary Figure S7

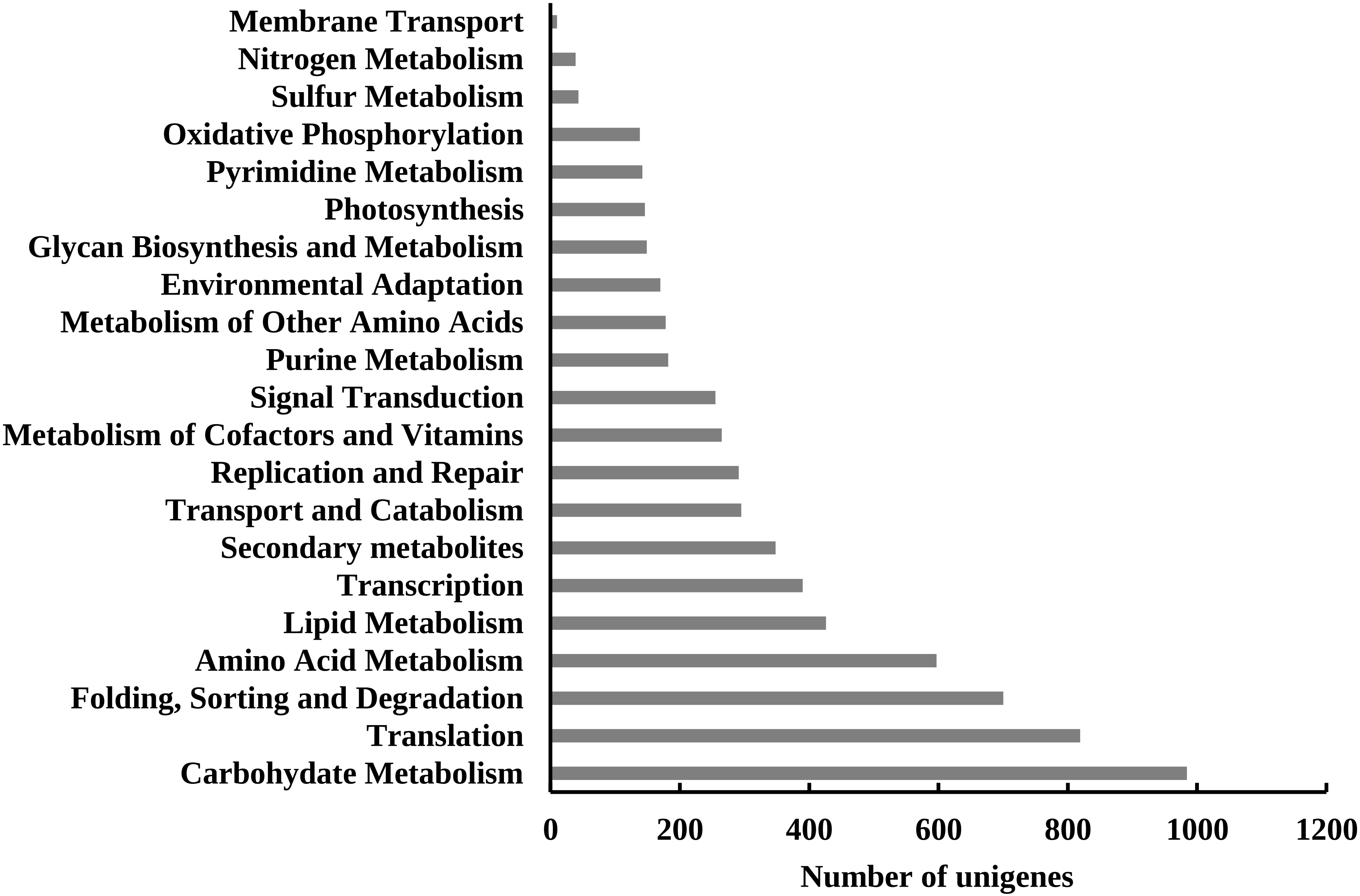

Supplementary Fig. S7. Distribution of unigenes into biological pathways using KEGG.

Supplementary Figure S8

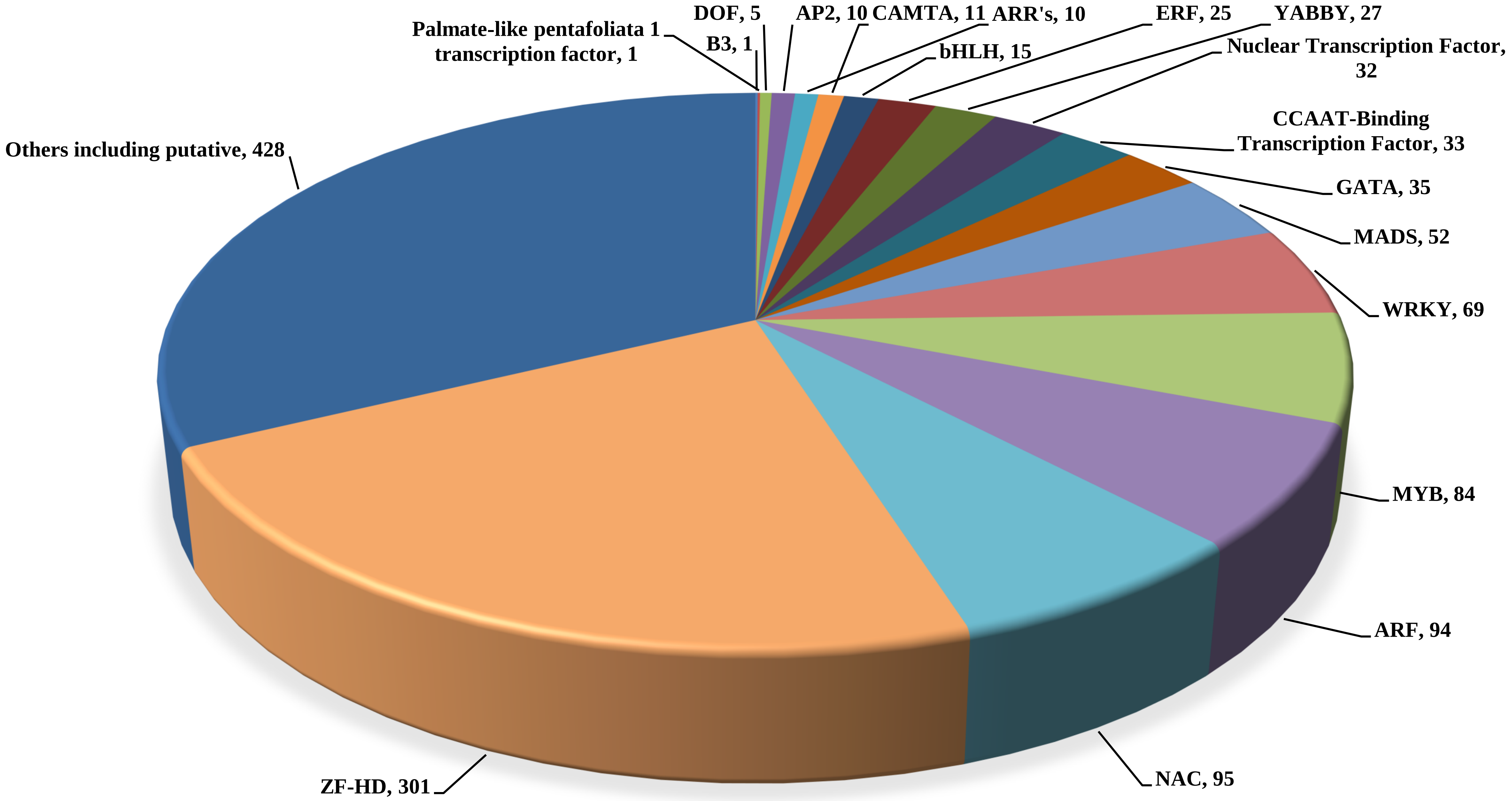

Supplementary Figure S8. Distribution of unigenes into transcription factor families.

Supplementary Figure S9

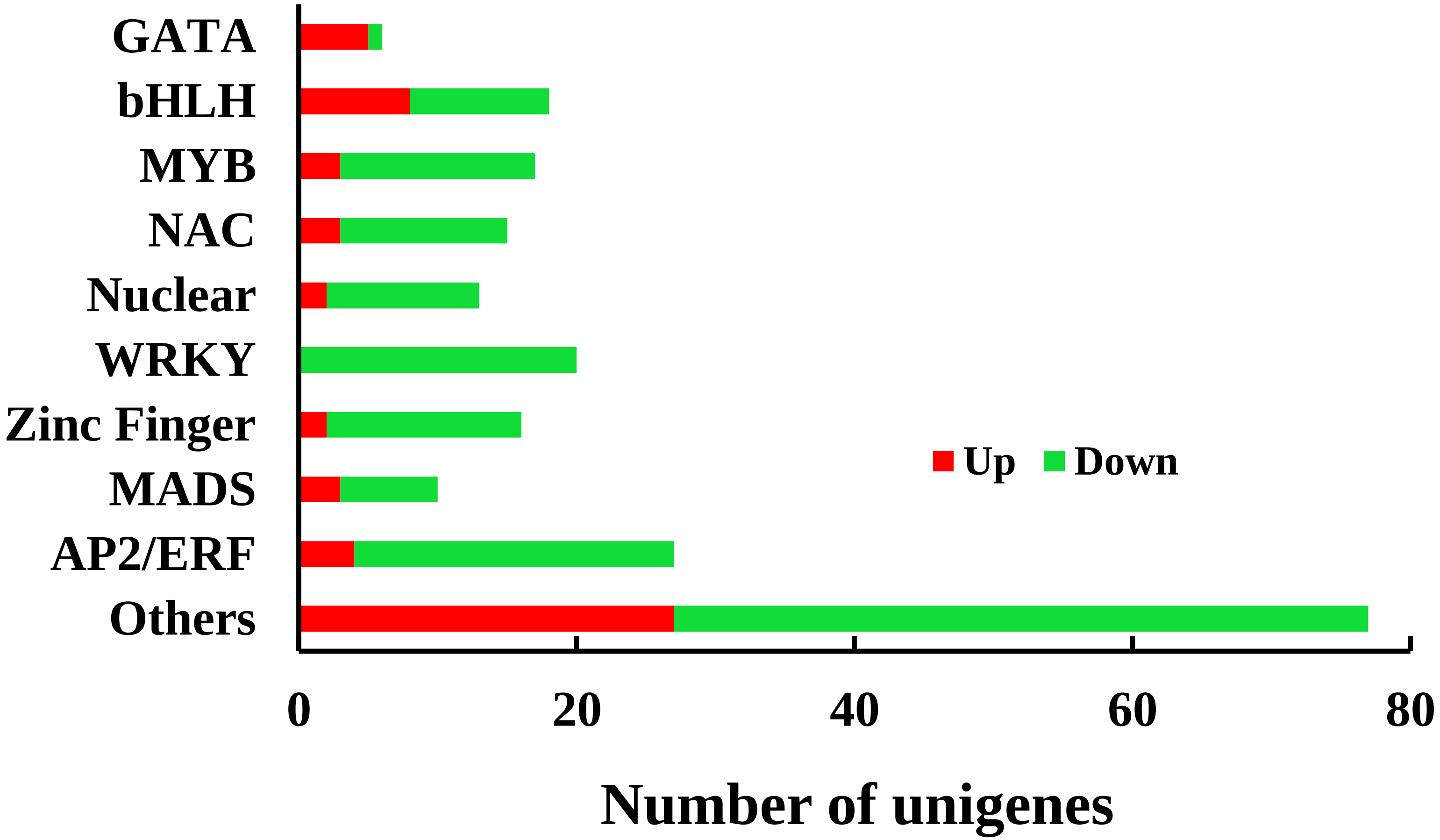

Supplementary Figure S9. Transcription factors differentially expressed in leaves of elevated CO_2 grown *Jatropha*.

Supplementary Figure S10

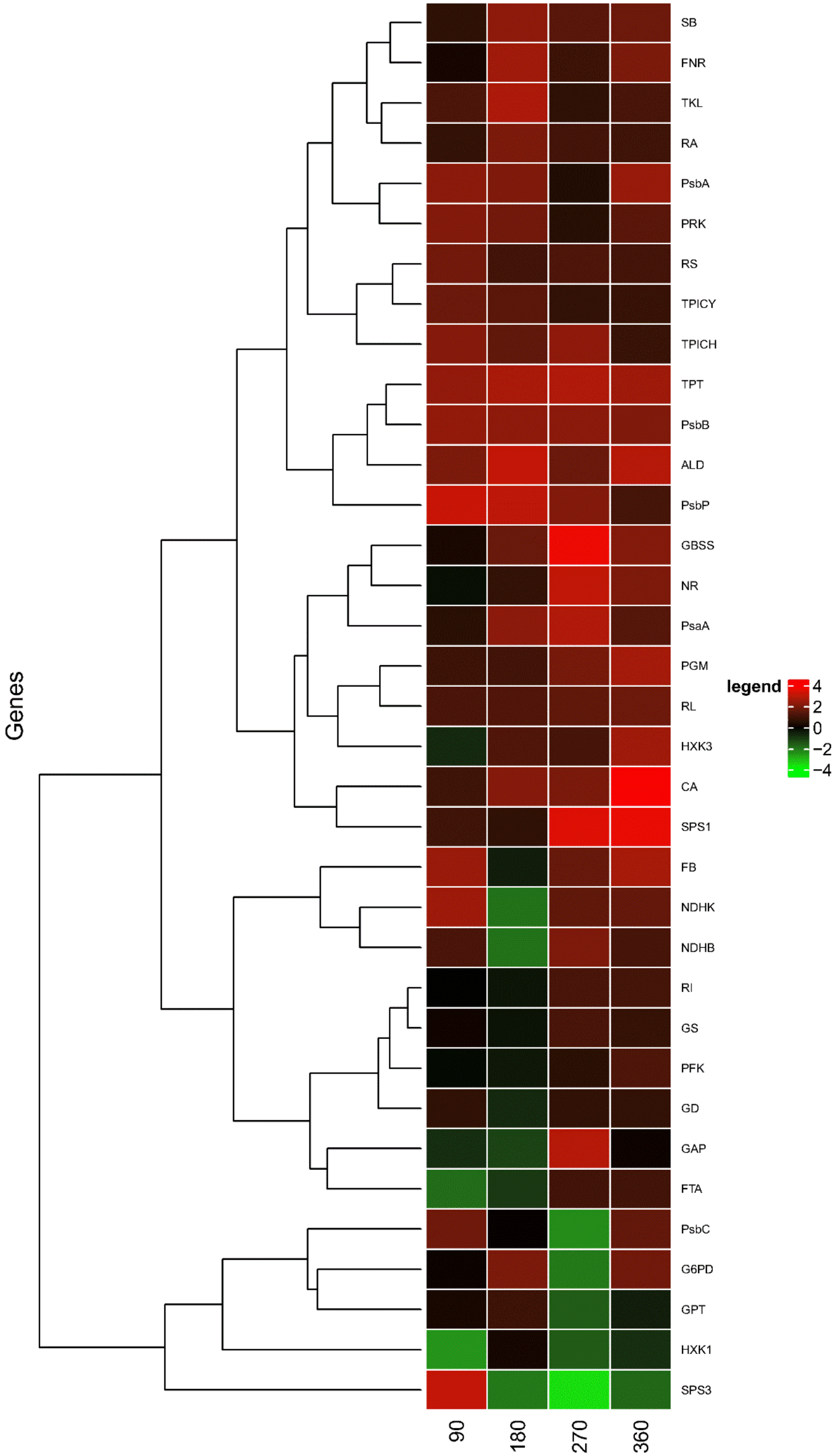

Supplementary Figure S10. A representative heat map of temporal expression pattern of 35 genes associated with photosynthesis and carbohydrate metabolism at four different time points as recorded in qRT-PCR analysis.

Supplementary Table S2. Assembly statistics of *Jatropha curcas* L. transcriptome under elevated CO_2.

Transcript Statistics	Sample A		Sample E	
	Contig	Transcripts	Contig	Transcripts
**K-mer**	49		53	
**% reads used for assembly**	86.58	97.05	83.69	97.14
**Number of transcripts identified**	44179	61779	28825	48755
**Maximum Contig Length**	8302	10713	6817	9793
**Minimum Contig Length**	200	200	200	200
**Average Contig Length**	828.2 ± 723.3	1,725.4 ± 1,185.9	1,008.7 ± 810.4	1,546.0 ± 1,004.2
**Median Contig Length**	409	3281	2304	3417
**Total Contigs Length**	3,65,88,305	10,65,94,308	2,90,75,500	7,53,73,695
**Total Number of Non-ATGC Characters**	11918	2680	8199	1728
**Percentage of Non-ATGC Characters**	0.033	0.003	0.028	0.002
**Contigs >= 200 bp**	44179	61779	28825	48755
**Contigs >= 500 bp**	25145	52438	18769	41010
**Contigs >= 1 Kbp**	11825	42537	10907	32316
**Contigs >= 10 Kbp**	0	2	0	0
**N50 value**	1187	2304	1522	2032

Supplementary Table S3. Reference assisted denovo assembly of both samples (A and E) with the *Jatropha* genome [*Jatropha curcas* cultivar: GZQX0401 RefSeq Genome; Genome version available in NCBI: JatCur_1.0, (http://www.ncbi.nlm.nih.gov/genome/Jatrophacurcas)]. The genome was downloaded from NCBI (Accession: GCA_000696525.1).

**Samples**	**A**	**E**
**Raw read count**	42693007	40849528
**Processed read count**	38692273	37131099
**Alignment percentage**	97.67	98.06
**Alignment percentage to gene models**	91.61	92.9
**Gene model covered**	21846	21431
**Transcripts expressed**	33782	33159
**Unaligned reads to genome**	562112	352822
**Number of transcripts identified**	1809	808
**Maximum Contig Length**	5189	3609
**Minimum Contig Length**	200	200
**Average Contig Length**	513.7 ± 438.5	459.4 ± 410.5
**Median Contig Length**	257	367.5
**Total Contigs Length**	9,29,307	3,71,229
**Total Number of Non-ATGC Characters**	140	71
**Percentage of Non-ATGC Characters**	0.015	0.019
**Contigs >= 200 bp**	1809	808
**Contigs >= 500 bp**	606	207
**Contigs >= 1 Kbp**	169	48
**Contigs >= 10 Kbp**	0	0
**N50 value**	617	513

Supplementary Table S8. List of primers for selected genes used for qRT-PCR analysis for the confirmation of gene expression. [*The gene IDs were assigned according to *Jatropha* genome version JatCur_1.0 (http://www.ncbi.nlm.nih.gov/genome/jatrophacurcas); F – Forward primer (5′-3′); R – Reverse primer (5′-3′)].

Description	Gene ID*	Primer Sequence
Carbonic anhydrase (CA)	105640386	**F** – CCGGCTTCGTCCACTTTAAG<br>**R** – CTGGCAATGTTTCGGACCAT
Sedoheptulose 1,7-bisphosphatase (SB)	105646676	**F** – TTGGGGAATCATTGCGGTTG<br>**R** – CTCCCATGCACATCAGAAGC
Fructose 1,6-bisphosphatase (FB)	105629115	**F** – GATGCACCAAAGCCACTTGA<br>**R** – GGGGATCTGTTACCACCACA
Sucrose phosphate synthase 1 (SPS1)	105633156	**F** – GTCTGATGTCATGCGGTTCC<br>**R** – TGTCATCCCTGTTCCCCATT
Granule bound starch synthase (GBSS)	105639084	**F** – ACTCTATGGTCCTCAGGCAG<br>**R** – GGTAGCAGGGAAGAAGAGCA
Glutamate dehydrogenase (GD)	105632353	**F** – AGCGAAAGGAGGGATTGGAT<br>**R** – GCCTCTCTACCCAGTGATCC
Glutamine synthetase (GS)	105631892	**F** – TTGTTGCCGAAGAGTGTTGG<br>**R** – ATGCCTTGTAGTGGGAGTCC
Nitrate reductase (NR)	105638879	**F** – GTGAAACGTGGCAAGTCTGT<br>**R** – GCAGTTGTTCATCATGCCCA
Hexokinase 3 (HXK3)	105631535	**F** – GGTTTGCATCCTTCACCTGG<br>**R** – TCATTGACCAAAGCTGCCAC
Rubisco large subunit (R_L)	7564870	**F** – TAAAACTTTCCAAGGGCCGC<br>**R** – AAATCAAGTCCACCGCGAAG
Rubisco small subunit (R_S)	105642030	**F** – CCGGCTTCGTCCACTTTAAG<br>**R** – TCCACATTGTCCAGTAGCGT
Rubisco activase (RA)	105647331	**F** – ATCTTGGGGATTTGGGGAGG<br>**R** – TGCGTAGTTCCACCCATTCT
F-type H+-type transporting ATPase subunit beta (FTA)	7564869	**F** – GTACCTGCGGACGATTTGAC<br>**R** – AACGATCTGAGGTTGGAGCA

Phosphofructokinase (PFK)	105632412	**F** – TGCTTCAGGATGTTGGGCTA<br>**R** – GCAAGGAGTGTGCAGAAGAC
Phosphoglucomutase (PGM)	105634080	**F** – GCTCGGTCTATGCCAACAAG<br>**R** – ACAGCCCATATGCCATCCTT
Ribose-5-PO_4 isomerase (RI)	105647678	**F** – AGTCTGGTATGGTTCTCGGC<br>**R** – ATCGACGTTGGGGTGAGAAT
Glyceraldehyde-3-PO_4 dehydrogenase (GAP)	105630249	**F** – AGCTCCTTCAAGAATCCCGG<br>**R** – TTGATGCTGCTACCTTGGGA
Ferredoxin-NADP reductase (PetH)	105635331	**F** – AGACCTGGCACATGGTCTTC<br>**R** – TGAGCAGACTCCCTTCACAA
Hexokinase 1 (HXK1)	105631535	**F** – AACCCTAGCTGGAGGCAAAT<br>**R** – TCAGTGGAAGATGCGATGAC
Triose phosphate/phosphate translocator (TPT)	105649590	**F** – GCTCTCTTTGCTTGCATTCC<br>**R** – ACATTGCCAACTGCATGTGT
Cytosolic triose phosphate isomerase (TPICY)	105647635	**F** – GCCGTCATCTGATGTTGTTG<br>**R** – CCGAGAATGACCCACAGAAT
Chloroplastic triose phosphate isomerase (TPICH)	105643783	**F** – TTGATGTTGTTGTCGCACCT<br>**R** – ACCCACTTGCAGCCAATATC
Sucrose phosphate synthase 3 (SPS3)	105633156	**F** – ACGGAGCTCTTGCTCACATT<br>**R** – TTCTTCCAAGCGAGTGTCCT
Photosystem (II), PsbP	105641916	**F** – ATACACGGATGCCAAGGAAG<br>**R** – GCTTGTCAGCCACAAACTGA
PsbC, chloroplast	7564849	**F** – GATCAACGTCTTGGGGCTAA<br>**R** – GGCGCATGGGTCATATATTC
PsbB, chloroplast	7564760	**F** – TCGAAGGGCAGAGTCAAAGT<br>**R** – AAGAGCAGAGCAAACGAAGC
PsbA, chloroplast	7564824	**F** – AGGCTGAGCACAACATCCTT<br>**R** – AATAACCATGAGCGGCTACG
PsaA, chloroplast	7564856	**F** – CAAGTGGTTTGGCCGATAGT<br>**R** – CATCTTGGAACCAAGCCAAT
Phosphoribulokinase, chloroplastic (PRK)	105631767	**F** – AAGTTTTGCCAACCCAACTG<br>**R** – CAGGGTAAGAGCAGGTGAGC

Glucose-6-phosphate dehydrogenase, chloroplastic (G6PD)	105641505	**F** – CACGTTTCAGTCCTTGCTCA<br>**R** – CATCCTGCAAAAAGACAGCA
Glucose-6-phosphate/phosphate translocator 1, chloroplast (GPT)	105645263	**F** – GAACCTGGGAAGGCAACATA<br>**R** – ATGTCACCGGATTCCTGAAG
Cytosolic aldolase (ALD)	105639139	**F** – TTCTGATGGAAAACCCTTCG<br>**R** – CGGGCACCTGCCTTATAGTA
Transketolase (TKL)	105643137	**F** – GCAATGTCAGGTTTGGTGTG<br>**R** – AAGCTTGCCAAGTGCTCAAT
NAD(P)H-quinone oxidoreductase subunit 2, chloroplastic (NDHB)	7564781	**F** – TGGGGCAAGCTCTTCTATTC<br>**R** – GCTTGAACCCGATTCCTACA
NAD(P)H-quinone oxidoreductase subunit K, chloroplastic (NDHK)	7564864	**F** – GTCCTAGACAAGCGGACCTG<br>**R** – CTTATCGACTCCCCGAACAG
18SrRNA	105629717	**F** – CCTGCGGCTTAATTTGACT<br>**R** – TTAGCAGGCTGAGGTCTC
